# Immunological landscape of human lymphoid explants during measles virus infection

**DOI:** 10.1172/jci.insight.172261

**Published:** 2024-07-25

**Authors:** Joshua A. Acklin, Aum R. Patel, Andrew P. Kurland, Shu Horiuchi, Arianna S. Moss, Emma J. DeGrace, Satoshi Ikegame, Jillian Carmichael, Shreyas Kowdle, Patricia A. Thibault, Naoko Imai, Hideki Ueno, Benjamin Tweel, Jeffrey R. Johnson, Brad R. Rosenberg, Benhur Lee, Jean K. Lim

**Affiliations:** 1 Department of Microbiology,; 2 Graduate School of Biomedical Sciences, and; 3 Department of Otolaryngology, Icahn School of Medicine at Mount Sinai, New York, New York, USA.

**Keywords:** Infectious disease, Virology, Cellular immune response

## Abstract

In humans, lymph nodes are the primary site of measles virus (MeV) replication. To understand the immunological events that occur at this site, we infected human lymphoid tissue explants using a pathogenic strain of MeV that expresses GFP. We found that MeV infected 5%–15% of cells across donors. Using single-cell RNA-Seq and flow cytometry, we found that while most of the 29 cell populations identified in the lymphoid culture were susceptible to MeV, there was a broad preferential infection of B cells and reduced infection of T cells. Further subsetting of T cells revealed that this reduction may be driven by the decreased infection of naive T cells. Transcriptional changes in infected B cells were dominated by an interferon-stimulated gene (ISG) signature. To determine which of these ISGs were most substantial, we evaluated the proteome of MeV-infected Raji cells by mass spectrometry. We found that IFIT1, IFIT2, IFIT3, ISG15, CXCL10, MX2, and XAF1 proteins were the most highly induced and positively correlated with their expression in the transcriptome. These data provide insight into the immunological events that occur in lymph nodes during infection and may lead to the development of therapeutic interventions.

## Abstract 2

Here, we evaluated measles infection in human lymphoid explants. We observed preferential infection of B cell subsets and a type I interferon driven host response.

## Introduction

Measles virus (MeV) is the most infectious human virus, with a reported R_0_ value of 12–18 (1–7). MeV outbreaks have largely been controlled with the advent of the 2-dose measles, mumps, and rubella (MMR) vaccine, yet MeV causes approximately 200,000 deaths annually, primarily among unvaccinated children in developing countries (8–10). However, recent surges in vaccine hesitancy have allowed MeV to reemerge in countries like the United States and the United Kingdom, where the MMR vaccine coverage has historically been high (11–14). With many global vaccination campaigns for MeV stalled because of the COVID-19 pandemic, the risk of MeV outbreaks globally continues to grow (15). Compounding these crises is the lack of any licensed antiviral that targets MeV once individuals are infected (16, 17).

MeV is a morbillivirus of the family Paramyxoviridae that is transmitted through the respiratory tract, where alveolar macrophages and dendritic cells are the initial cellular targets of infection (18, 19). These infected immune cells then traffic to the draining lymph nodes, where the virus replicates rapidly in lymphocytes that express the entry factor CD150/SLAMF1 (20–23), followed by egress through lung epithelium that is mediated by basolateral expression of the Nectin-4 receptor (23–27). MeV is also known for causing immune amnesia through the depletion of CD150^+^ B and T cells in both primary and secondary lymphoid organs, increasing the morbidity and mortality rates from secondary infections with common childhood pathogens (28–35). Immunological amnesia following MeV infection has been shown to markedly reduce the antibody repertoire toward common childhood pathogens, such as the human parainfluenza viruses, respiratory syncytial virus, coronaviruses, and cytomegalovirus (36). Given that immune responses are primarily generated in the draining lymph node, and that this site is a critical launching pad for MeV infections, understanding virus/host interactions at this site is paramount for identifying factors that shape disease progression.

Modern reanalysis of early work on immunological amnesia caused by measles implicates T cells because of a delayed type I hypersensitivity response to tuberculin antigen (37, 38). In vitro characterization of MeV infection in primary lymphocytes revealed that B cells are the most extensively infected population of lymphocytes, consistent with their high CD150 expression (39). Characterization of MeV infection in PBMCs from both humans and macaques further demonstrated the propensity of MeV for lymphocytes and that MeV infection is biased toward naive B cells, memory B cells, and memory T lymphocytes, which was subsequently validated in PBMCs derived from MeV-infected children (18, 34, 40, 41).

While these studies provide insights into MeV infection of lymphocytes, they do not examine infection within the complex architecture of secondary lymphoid tissue. The draining lymph nodes are organized with high-density B cell follicles surrounded by T cell zones (42), which may be important to determine the in vivo cellular susceptibility as well as the kinetics of lymph node infection. Studies in macaques have examined the geographic distribution of infected cells within secondary lymphoid tissues and have identified that the majority of infection is established within B cell follicles (41). Further, these studies recapitulated the heightened susceptibility to infection among memory but not naive lymphocyte subsets within secondary lymphoid organs (41). Studies using human tonsil explants have also been conducted, where lymphoid tissue structures and native cell ratios are intact, which found that B cells are a preferential target of MeV infection and that memory T cells were more extensively infected compared with naive T cell subsets (43, 44). In this study, we revisited this human lymphoid explant model using a GFP-expressing pathogenic isolate of MeV, which has been shown to mimic human clinical outcomes in nonhuman primates, commonly referred to as a wild-type isolate (45). Our findings both confirm and extend our understanding of cellular susceptibility to MeV in humans. While a similar analysis of MeV-infected airway epithelium has been conducted (46), we present transcriptional signatures of MeV-infected lymphocytes with single-cell resolution in human lymphoid tissue explants and link these transcriptional signatures with translated products in the proteome.

## Results

### MeV replicates efficiently in human lymphoid tissues ex vivo.

**Figure 1:**
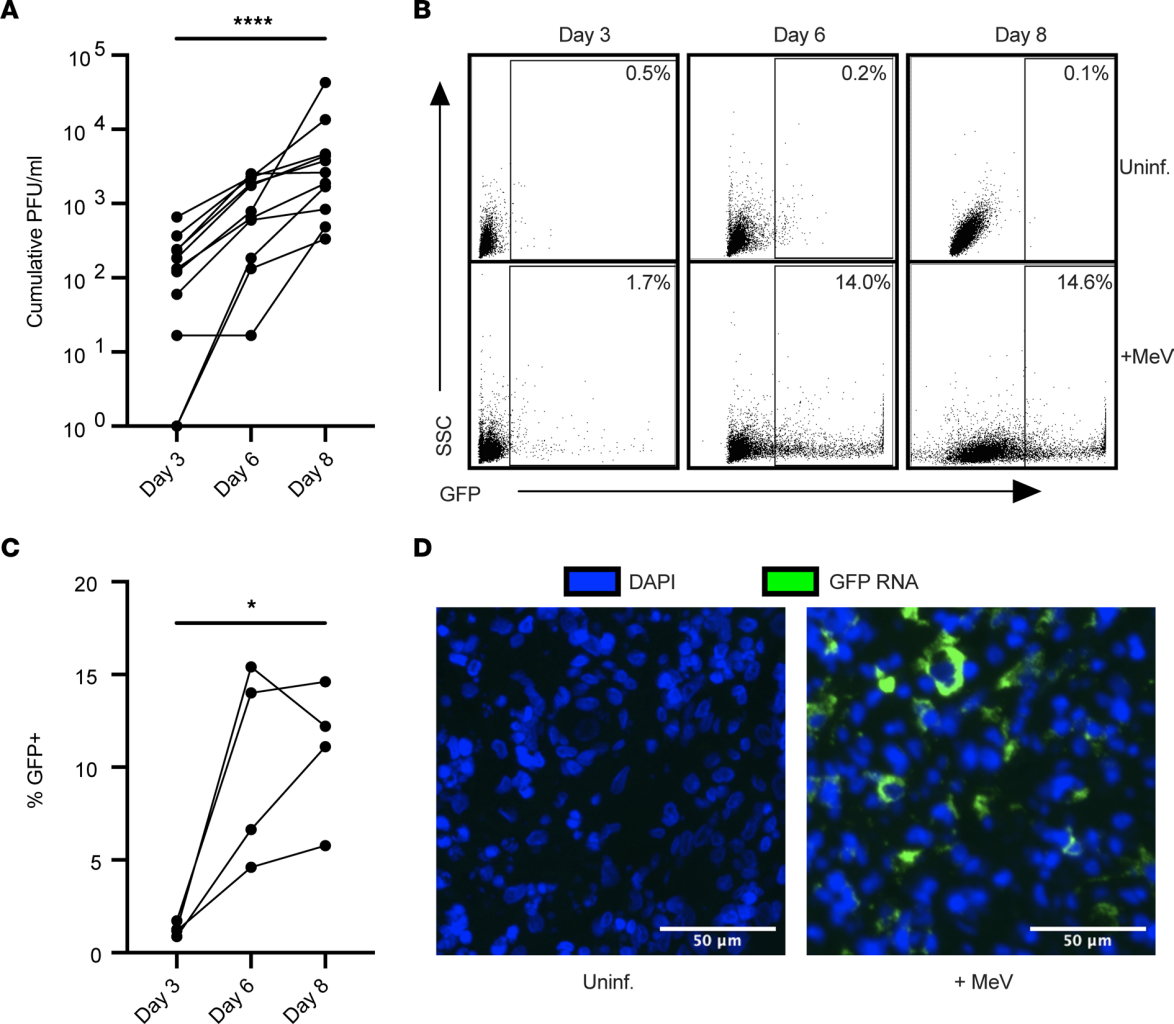
MeV productively infects human lymphoid tissue explants. Tonsil tissues (*n* = 11) were infected with MeV-GFP. Cumulative viral plaque-forming units (PFU/mL) were quantified from supernatants collected on days 3, 6, and 8 after infection by plaque assay (**A**). Representative flow plots quantifying infection (GFP) are shown for 1 donor (**B**) and quantified across 4 donors over time (**C**). In situ hybridization for GFP RNA (green) on paraffin-embedded tissues collected on day 8 after infection compared with a donor-matched uninfected control (**D**). Nuclei were counterstained with DAPI (blue). Scale bars represent 50 μm. Significance was determined by 1-way ANOVA using Friedman’s test with Dunnett’s multiple-comparison test. * indicates *P* < 0.05, and **** indicates *P* < 0.0001.

To evaluate how MeV infection proceeds within human lymphoid tissue, we infected human tonsil tissues ex vivo, the most accessible lymphoid tissue for laboratory use. Tissue samples from routine, noninflamed tonsillectomies were infected with a pathogenic isolate of MeV (IC323) that expresses GFP (MeV-GFP) as previously described (44, 45, 47). While this model lacks a functional lymphatic system, and thus does not exactly mimic the way that MeV enters the draining lymph node during human infections, it benefits from retaining the 3-dimensional tissue architecture of human lymph tissue. To assess the extent to which MeV could replicate in human lymphoid tissues across 11 donors, we collected culture supernatants at days 3, 6, and 8 after infection and measured virus production by fluorescence plaque assay. As shown in Figure 1A, viral titers increased for all donors, with approximately 2.5 log increase over the 8-day culture.

To assess the extent of infection within the tissues and to further verify productive infection, we measured the frequency of GFP^+^ cells over time by flow cytometry. As shown in Figure 1B, for 1 representative donor, the percentage of GFP^+^ cells increased over time, while no GFP signal was detected in the uninfected condition. Quantification across 4 donors revealed a frequency of MeV-infected cells that ranged between 5% and 15% of live cells by day 8 after infection (Figure 1C). Further, RNA transcripts for GFP were readily detected within the tissues through in situ hybridization (Figure 1D). Together, our data establish that human tonsils infected ex vivo are susceptible to MeV without any stimulation or infection enhancers, providing us with a robust model system to further characterize MeV-infected cells.

### scRNA-Seq of MeV-infected tissues reveals broad cellular susceptibility.

**Figure 2:**
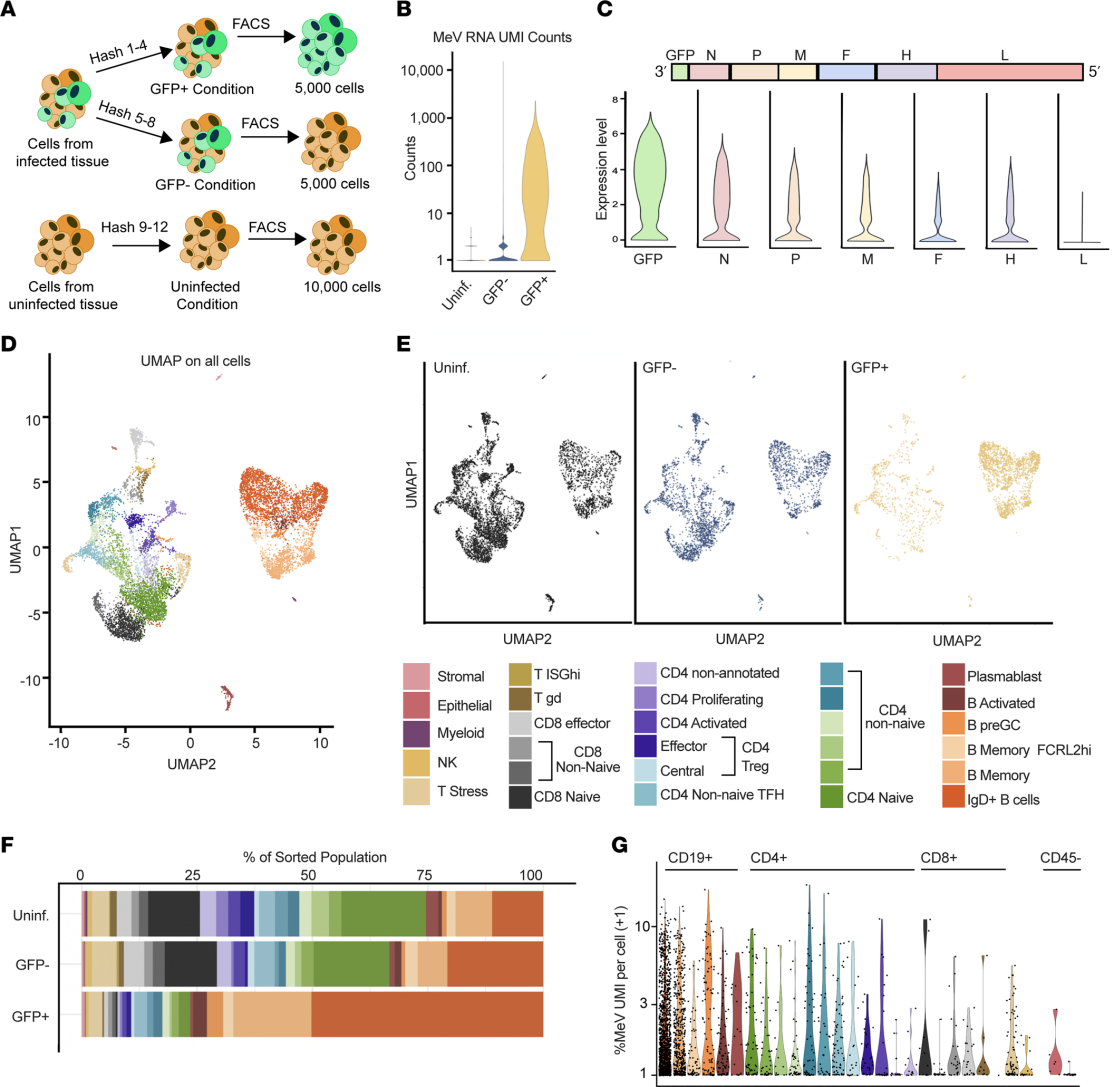
scRNA-Seq identifies 29 unique cell populations in tonsils susceptible to MeV. Tonsil tissue from MeV-GFP–infected and uninfected explants on day 8 from 1 donor were sorted for scRNA-Seq. Schemata of scRNA-Seq workflow are shown (**A**). Cells from the infected condition were sorted and hash-labeled into GFP^+^ and GFP^–^ groups. Uninfected GFP^–^ cells were sorted from a donor-matched uninfected control. A total of 5,000 GFP^+^ cells, 5,000 GFP^–^ cells, and 10,000 uninfected cells were encapsulated for sequencing. MeV RNA unique molecular identifiers (UMIs) were quantified for quality control and filtering (**B**). Normalized expression of each MeV transcript in infected cells was quantified and shown as violin plots ordered from 3′ to 5′ in the MeV genome (**C**). Canonical correlation analysis was conducted on all groups (combined), and individual clusters were functionally annotated (see also Supplemental Figure 1). Clusters were visualized by UMAP (**D**) and then split into conditions based on captured hashing oligonucleotide sequences for further analysis (**E**). The frequencies of each cell cluster identified in **E** were calculated for each group, and quantification is shown in **F**. The percentage of the transcriptome that is MeV RNA is shown in **G**, with a +1 pseudocount artificially added to the values for display on a log_10_ axis. All cluster annotations are labeled by the color legend shown.

Given the high percentage of GFP^+^ cells detected, we sought to sort and analyze infected cells using single-cell RNA-Seq (scRNA-Seq). To do this, we generated single-cell suspensions from MeV-infected and donor-matched uninfected tonsil tissue on day 8 after infection, the time point at which the maximum number of infected cells was observed. We sorted GFP^+^ (infected) and GFP^–^ (bystander) cells from the MeV-infected condition, as well as the GFP^–^ cells (uninfected) from the uninfected condition for scRNA-Seq using a workflow shown in Figure 2A. We validated the quality of sequencing across the 3 groups by quantifying unique molecular identifier (UMI) counts, unique genes captured, and the representation of mitochondrial transcripts (Supplemental Figure 1, A–C; supplemental material available online with this article; https://doi.org/10.1172/jci.insight.172261DS1). As expected, only cells from the infected group had appreciable MeV transcripts (Figure 2B). Among the infected cells, we examined the expression levels of viral transcripts as a final confirmation of infection status. Infected cells showed a transcriptional gradient of viral genes from 3′ to 5′, consistent with the phenomenon of run-off transcription that occurs for paramyxoviruses (Figure 2C).

Following data integration, we annotated constituent cell clusters across conditions based on immune cell reference data and supervised differential marker gene analysis (see Methods). Using this strategy, we identified 29 distinct populations of cells (Figure 2D), with the vast majority belonging to either T or B cell subsets (annotation strategy shown in Supplemental Figure 1D). Interestingly, GFP^+^ transcripts were identified in all 29 populations, albeit to different levels in each population, spanning subpopulations of CD4^+^ and CD8^+^ T cells, B cells, tonsillar epithelium, and tonsillar stromal cells (Figure 2E). We were surprised by this finding and assessed the expression of *SLAMF1*, the gene encoding the canonical MeV receptor CD150, in each cluster. As shown in Supplemental Figure 1E, the detection of *SLAMF1* in our dataset was not robust. However, we detected higher levels among B cell populations, particularly among activated B cells, which is consistent with previous reports (39, 41, 43). We next quantified the frequency of each of these populations within the uninfected, bystander, and infected cell–sorted sample groups and found that, despite T cells comprising the largest population of cells in human tonsils (as shown in uninfected and bystander groups), they were not the majority among the MeV-infected cells. Instead, IgD^+^ B cells were overrepresented within the pool of GFP^+^ cells (Figure 2F). To assess the extent to which the transcriptome is co-opted for viral gene expression, we quantified the percentage of UMI counts that mapped to MeV UMIs for each identified cell population. As shown in Figure 2G, MeV transcripts constituted approximately 0.93% of all transcripts for each cluster on average (calculated using the median % viral UMI per cluster). One possible explanation for differences in the frequency of cell clusters among the GFP^+^ condition would be that cells had either proliferated or died during or because of preferential infection of various cell subsets found within the tissue. To assess this possibility, we evaluated the B cell clusters for gene signatures associated with proliferation. As shown in Supplemental Figure 1, F and G, while we observed high pathway scores for S phase and G_2_/M phase among CD4^+^ cells (annotated as proliferating in Figure 2), we did not observe any elevation within any B cell cluster, regardless of infection status. However, as this analysis was limited to a single donor at a single time point, we can only conclude that MeV has a wide cellular tropism within the lymphoid explants and suggest that IgD^+^ B cells are the primary target.

### B cells are preferential targets of MeV infection in lymphoid tissue explants.

**Figure 3:**
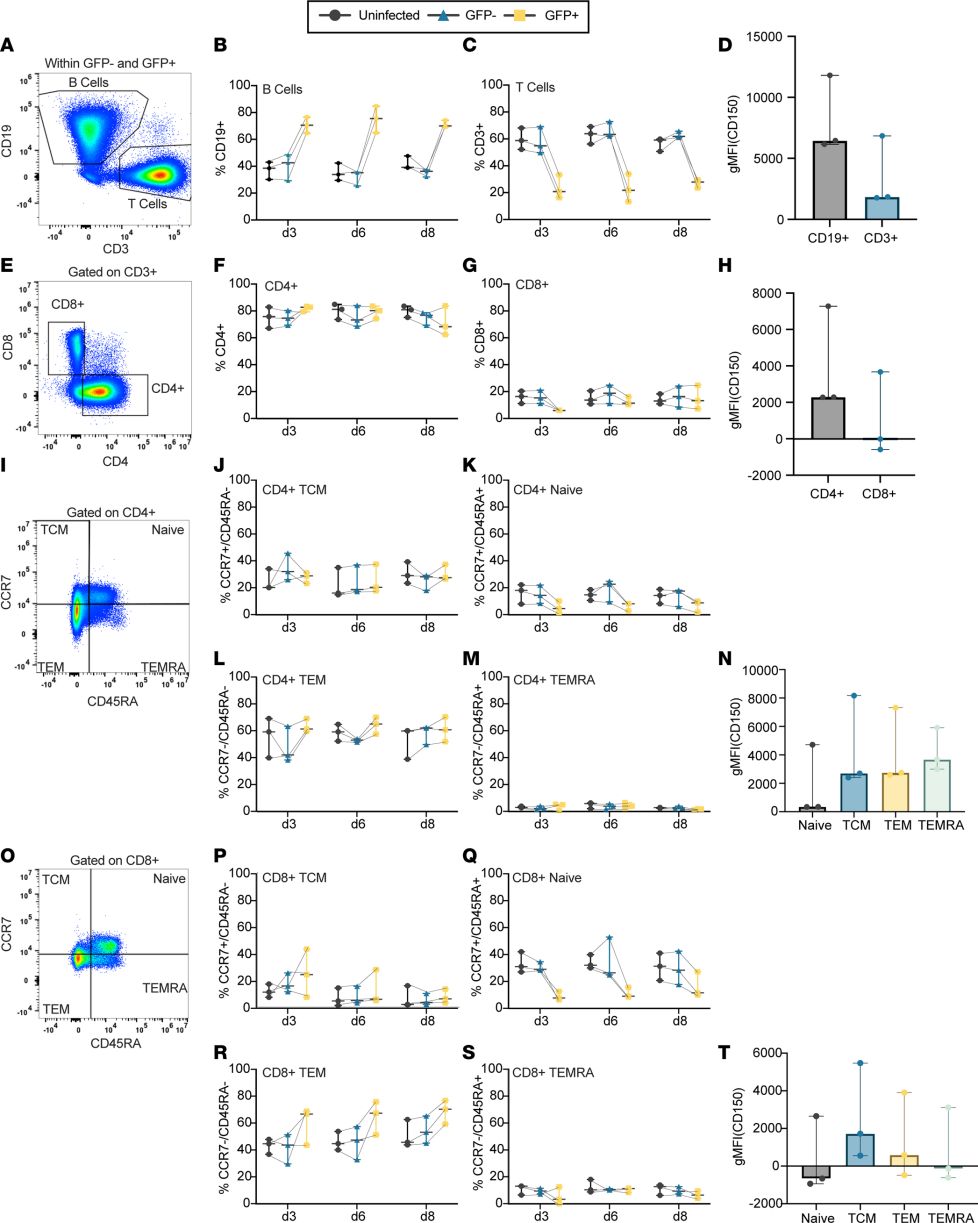
MeV preferentially infects B cells and is restricted among naive T cells. Cells from MeV-GFP–infected and donor-matched uninfected tissues were collected at days 3, 6, and 8 after infection and were then immunophenotyped by flow cytometry (*n* = 3; gating schemata in **A**, **E**, **I**, and **O**). The frequency of CD19^+^ B cells (**B**) and CD3^+^ T cells (**C**) among all CD45^+^ cells are quantified and compared over time for GFP^+^ cells, GFP^–^ bystander cells, and donor-matched uninfected controls. The geometric mean fluorescence intensity (gMFI) of surface CD150 among CD19^+^ and CD3^+^ cells is compared (**D**). Susceptibility to infection among CD4^+^ (**F**) and CD8^+^ (**G**) populations is shown, with CD150 expression compared between populations (**H**). Naive (CD45RA^+^CCR7^+^), TCM (CD45^+^CCR7^–^), TEM (CD45RA^–^CCR7^–^), and TEMRA (CD45RA^+^CCR7^–^) populations are quantified and compared among CD4^+^ cells (**I**–**M**) and CD8^+^ cells (**O**–**S**). CD150 expression is compared among CD4^+^ (**N**) and CD8^+^ (**T**) subpopulations. For all immunophenotyping panels, significance was determined by 2-way ANOVA using the Geisser-Greenhouse correction with Tukey’s multiple-comparison test. For panels **D** and **H**, significance was determined by Wilcoxon’s matched pairs signed rank test. For panels **N** and **T**, significance was determined by 1-way ANOVA using Friedman’s test with Dunnett’s multiple-comparison test. For all plots, the median and the 95% confidence interval are shown.

While the scRNA-Seq analysis suggested that differences in susceptibility to MeV may exist within the lymphoid tissue explants, these data evaluated only a single time point for a single donor. To define MeV infection across donors and over time, we immunophenotyped major cell subsets identified in the scRNA-Seq dataset by flow cytometry (*n* = 3). We first quantified the frequency of MeV-infected B and T cells and compared these frequencies to their frequency among GFP^–^ bystander cells (from the infected condition) and donor-matched, uninfected cells (gating schemata in Figure 3A). For ease of data interpretation, we also show the frequencies of GFP over time within cell populations discussed in this section in Supplemental Figure 2 and present a complete gating schema for all analyses in Supplemental Figure 3. As shown in Figure 3B, there was a significantly higher frequency of CD19^+^ B cells among the GFP^+^ cells compared with their frequency in the uninfected or bystander populations. This difference was established by day 3 and maintained across the 8-day culture. Conversely, we observed a decrease in the frequency of CD3^+^ T cells (Figure 3C). Evaluation of CD150 expression by flow cytometry showed a trend toward higher levels of CD150 on CD19^+^ B cells compared with CD3^+^ T cells. While this did not reach statistical significance, this trend is consistent with the higher CD150 transcript counts observed in B cell populations compared with T cell populations by scRNA-Seq (Figure 3D and Supplemental Figure 1E).

We next asked whether the preference for B cell infection was driven by a specific B cell subset, or if all B cells were more susceptible to infection. To test this, we subset B cells based on CD38 and CD27 expression (gating schemata shown in Supplemental Figure 4A). To discriminate between susceptibility differences shared among all B cells and those that are subtype specific, we calculated the frequency of each B cell subset as a percentage of the total B cell pool among both GFP^+^ B cells from infected tonsil explants, as well as among bystander and uninfected B cells. We found essentially no differences in infection based on CD38/CD27 expression (Supplemental Figure 4, B–E). The lack of differences in infection between subsets is also consistent with the relatively stable expression of CD150 among these populations (Supplemental Figure 4F). Given the dramatic increase in the IgD^+^ B cell cluster observed among GFP^+^ cells in the scRNA-Seq (Figure 2F), we next assessed if IgD status conferred heightened susceptibility to infection among B cells across time. We recapitulated the finding that IgD^+^ cells are more frequent among the GFP^+^ population than among bystander and uninfected cells (Supplemental Figure 4G). However, when examined among CD19^+^ cells in each group, we found no evidence of preferential infection based on IgD status (Supplemental Figure 4, H and I). Likewise, CD150 expression was not different between IgD^+^ and IgD^–^ cells at day 6 (Supplemental Figure 4J). These data show that while B cells are more susceptible to MeV infection, this is most likely not driven by any individual subset of B cells.

We also evaluated the susceptibility of T cell subpopulations by examining the frequency of CD4^+^ and CD8^+^ cells among GFP^+^, bystander, or uninfected cell subsets. As shown in Figure 3, E–G, we identified no differences in susceptibility between helper (CD4^+^) and cytotoxic (CD8^+^) designations, consistent with the lack of differences in CD150 expression, which was generally low, between CD4^+^ and CD8^+^ T cells (Figure 3H).

### Reduced susceptibility of naive T cell subsets in human lymphoid tissue.

Previous reports have shown that mature (CD45RA^–^) T cell subsets are more susceptible to MeV infection than naive (CD45RA^+^) T cells (34, 40, 41). To assess this in our model, we evaluated the frequency of naive T cells (CD45RA^+^CCR7^+^), as well as non-naive T central memory (TCM; CD45RA^–^CCR7^+^), T effector memory (TEM; CD45RA^–^CCR7^–^), and T effector memory RA^+^ (TEMRA; CD45RA^+^CCR7^–^) subsets among CD4^+^ and CD8^+^ T cells from the GFP^+^, GFP^–^ (bystander), and uninfected groups. As shown through Figure 3, I–T, we observed reduced MeV infection in CD4^+^ and CD8^+^ naive T cells compared with the non-naive subsets, which were less frequent in the GFP^+^ population than in the bystander or uninfected groups, particularly at earlier time points. Evaluation of the non-naive subsets did not reveal an increased frequency among GFP^+^ cells, suggesting they were no more likely to become infected than their proportion in the culture. Assessment of CD150 expression on these T cell subsets shows that naive T cells trended toward less CD150 expression than mature subsets. These differences were not significant, suggesting that CD150 expression alone does not explain these trends in susceptibility. Last, we hypothesized that the proximity of follicular CD4^+^ T cells to highly susceptible B cells in the follicle could affect the susceptibility of these CD4^+^ cells to infection. To test this, we evaluated the frequency of MeV-infected cells among CD4^+^CD45RA^–^ T cells based on CXCR5 expression. As shown in Supplemental Figure 5, we found no difference in susceptibility based on CXCR5 status. Taken together, these data indicate a reduced susceptibility of naive T cells to MeV that is largely independent of CD4 or CD8 status, CD150 expression, and CXCR5 expression.

### MeV induces a canonical ISG response in both B and T cell transcriptomes.

**Figure 4:**
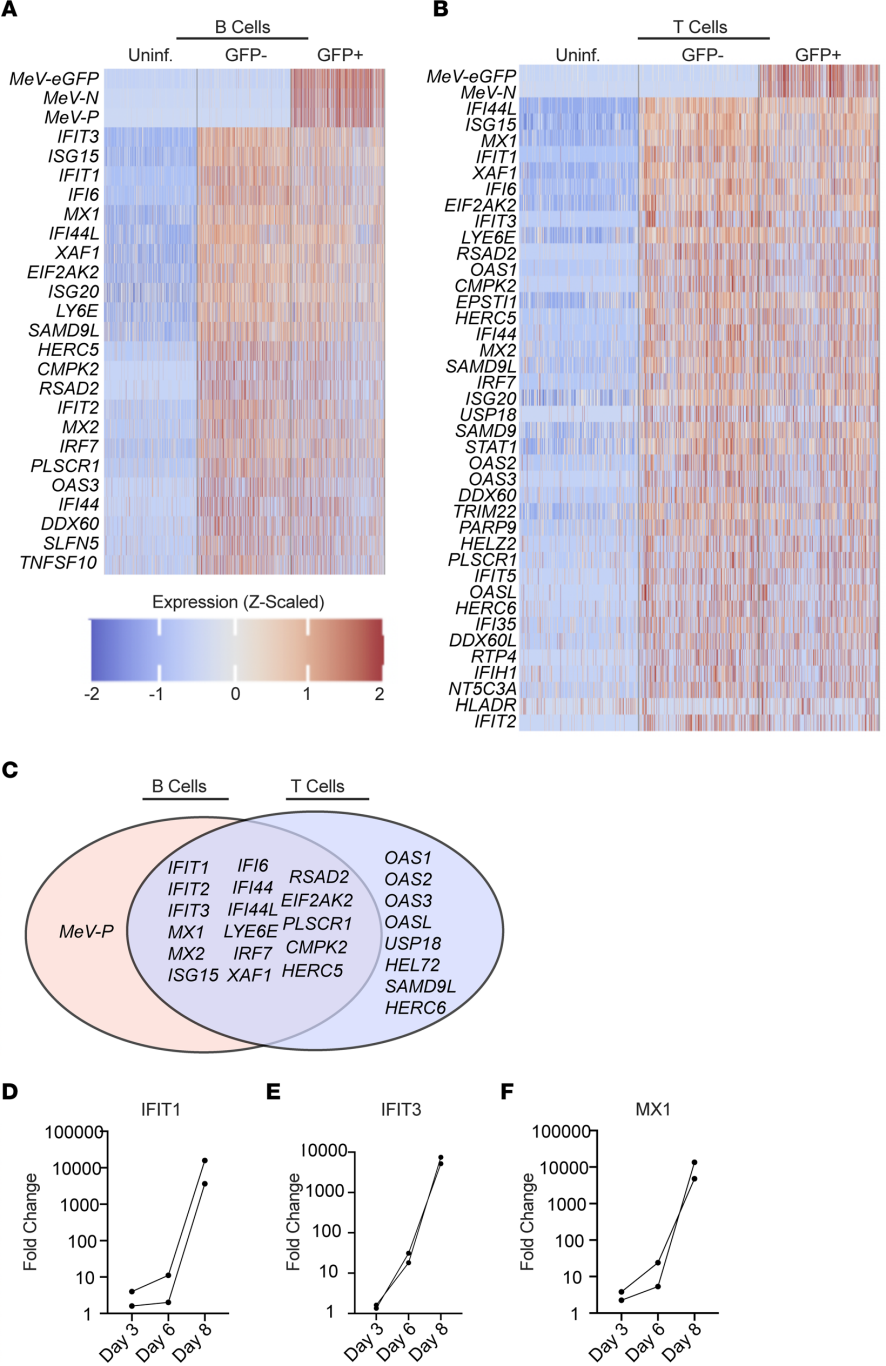
Host response to MeV in lymphoid tissue is dominated by a type I interferon response. All B and T cell clusters from the scRNA-Seq analysis were combined with stochastic downsampling. Differential gene expression analysis was conducted with EdgeR, and expression of the most significant genes is shown for B cells (**A**) and T cells (**B**). Statistical thresholds for significant differential gene expression were set at Benjamini-Hochberg–adjusted *P* < 0.0001 and absolute log fold-change (LogFC) > 1.58. Genes that were significant in either the infected/uninfected or the bystander/uninfected comparison were plotted. Genes significantly induced during infection were compared between B and T cells (**C**). MeV-infected tonsil explants were collected for RNA extraction and analysis by qRT-PCR. RNA was examined for the expression of *IFIT1* (**D**), *IFIT3* (**E**), and *MX1* (**F**). The FC in expression levels relative to uninfected controls is shown, with the expression of each ISG normalized to the expression of *GAPDH* (ΔΔCT method).

Given that the most striking susceptibility differences to MeV infection were observed between B and T cells, we next asked whether the host response to infection among these cell types could contribute to these differences in susceptibility. To test this, we randomly sampled an equal number of B cells or T cells from the uninfected, bystander, and GFP^+^ groups from the scRNA-Seq data and conducted differential gene expression analysis. As expected, the most significantly induced genes among the infected cells were MeV genes and GFP transcripts (Figure 4, A and B). Following the viral genes, the most significantly upregulated transcripts among GFP^+^ B and T cells were associated with a canonical interferon (IFN) signature. This pattern of IFN induction was strikingly similar among bystander cells, which were GFP^–^ (thus not containing viral transcripts). We detected expression of the edited MeV IFN antagonist transcript, *V*, in GFP^+^ cells but were unable to make meaningful comparisons in the expression of these transcripts among infected cell clusters because of the low read coverage at the p-editing site, where nontemplated nucleotide insertion distinguishes *V* transcripts from the more abundant *P* mRNAs (Supplemental Figure 6). To directly compare the host response in infected B and T cells, we constructed a Venn diagram of significantly induced genes in each group. As shown in Figure 4C, we found a highly conserved response between both cell types, among which *IFIT1*, *IFIT2*, *IFIT3*, *MX1*, *MX2*, *XAF1*, and other canonical interferon-stimulated genes (ISGs) were shared. While the only gene found to be uniquely induced in infected B cells as compared with infected T cells was the MeV *P* gene, T cells were found to have induced additional ISGs that were not significant in B cells, including *OAS1*, *OAS2*, *OAS3*, *OASL*, *USP18*, *HELZ2*, *SAMD9L*, and *HERC6*. This unique pattern of gene expression may be biologically meaningful or instead a consequence of strict statistical thresholding. Careful and directed comparative analyses using protein-based approaches will need to be conducted to confirm the relevance of these differences. To further validate this IFN signature, we conducted quantitative reverse transcription PCR (qRT-PCR) from the tissues of 2 additional tonsil donors over time. We evaluated the expression of *IFIT1*, *IFIT3*, and *MX1*, 3 of the highly expressed type I ISGs from the scRNA-Seq analysis. As shown in Figure 4, D–F, we found potent induction of all 3 genes by day 8 after infection, the time point of scRNA-Seq. Taken together, these data suggest MeV induces a potent IFN response at the transcriptional level in both infected and bystander B and T cells, with no notable differences that account for the increased susceptibility of B cells.

### MeV induces an IFN-driven response in B cells at the protein level.

**Figure 5:**
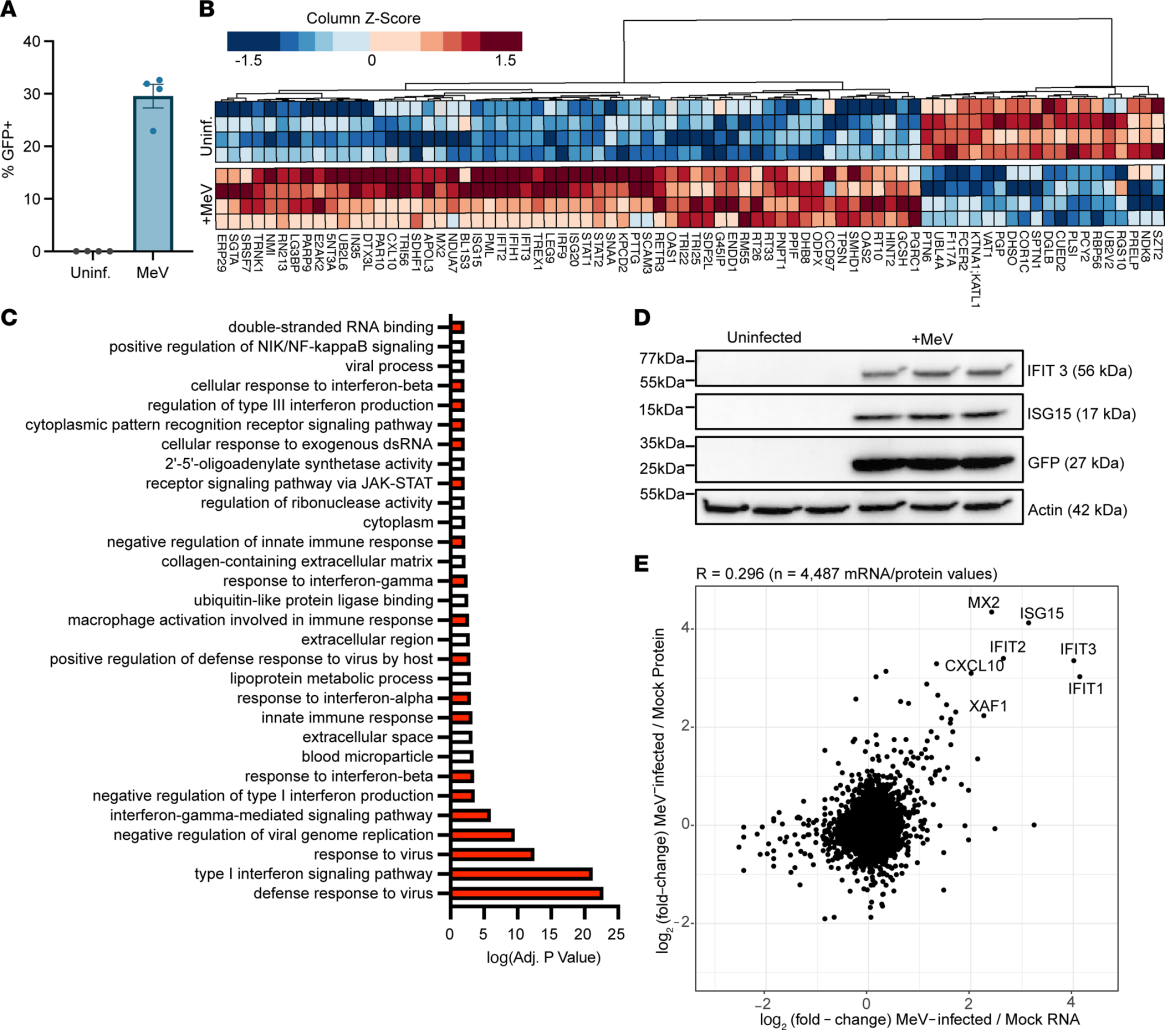
Proteins involved in a type I IFN response are potently upregulated in response to MeV. Raji-DCSIGNR cells were infected with MeV-GFP (MOI = 0.1) or left uninfected for 72 hours before processing for protein abundance mass spectrometry (*n* = 4). Infection was confirmed by quantifying GFP expression by flow cytometry (**A**). The most significantly dysregulated proteins (columns) for each sample (rows) were visualized, with clustering based on protein expression (**B**). GO analysis was conducted, and the most significant functional terms were visualized. Terms were given a positive value if the term was upregulated during infection or a negative value if downregulated. Red bars indicate involvement in antiviral responses (**C**). Validation of IFIT3 and ISG15 upregulation in infected Raji cells was conducted by Western blot (**D**). Correlation of the transcriptome (pseudobulked B cell supercluster from Figure 4) and proteome (Raji cells, Figure 5) was conducted. Values that had a log_2_FC > 2 in both the proteome and transcriptome were labeled (**E**). For the box-and-whisker plot, the mean with SEM is shown.

Since we identified a potent IFN signature in response to MeV at the transcriptional level, we next asked whether the corresponding proteins were expressed. As B cells were preferential targets for MeV, we utilized Raji cells where we could carefully define the protein level response to infection in a uniform cellular population. Raji cells were infected with MeV at an MOI of 0.1 for 72 hours, and infection was confirmed through GFP expression (Figure 5A). Infected and uninfected Raji cells were lysed, trypsin-digested, and analyzed by quantitative mass spectrometry. Protein abundance was quantified in each sample relative to uninfected samples. We next conducted differential expression analysis to quantify altered protein expression during MeV infection as compared with the uninfected condition. As shown in Figure 5B, MeV-infected B cells had higher expression of ISGs, consistent with our scRNA-Seq data.

To functionally annotate the significantly dysregulated proteins, we next conducted a gene ontology (GO) analysis. Significant GO terms are shown in Figure 5C, where the directionality of the response is artificially shown based on a positive (upregulated) or negative (downregulated) transformation of the adjusted *P* value for that term. We found that the most significantly upregulated pathways in MeV-infected cells were involved in antiviral signaling or IFN biology (colored in red; Figure 5C). To further validate these results, we selected IFIT3 and ISG15, 2 of the most highly upregulated proteins identified in the proteomics data, to examine by Western blot. Like the GFP signal, we found that both IFIT3 and ISG15 were expressed only in MeV-infected Raji cells (Figure 5D). Given that the transcriptomic analysis was conducted in primary human lymphoid tissue, while the proteomic analysis was conducted in a B cell line, we wanted to identify the significantly upregulated hits that were identified in both systems. To do this, we compared the MeV transcriptional signature among the pseudobulked B cell cluster (containing stochastic sampling of each of the B cell clusters) from lymphoid explants to the significant proteins identified in the proteome of infected Raji cells. As shown in Figure 5E, the most highly upregulated hits from this correlation analysis were ISGs, dominated by *MX2*, *ISG15*, *IFIT1*, *IFIT2*, *IFIT3*, *CXCL10*, and *XAF1*. To further characterize this host response, we also compared the transcriptome and proteome of infected Raji cells. Raji cells were infected as in the MS experiment but collected for bulk RNA sequencing. As shown in Supplemental Figure 7, we identified a conserved set of ISGs that were robustly and significantly upregulated at both the RNA and protein level. In addition, we noted that the expression of the FC-epsilon receptor was significantly downregulated. Taken together, our integrated approach of assessing the host response to infection at both the transcriptional and protein level reveals a potently induced IFN signature that is conserved between distinct infection systems.

## Discussion

MeV pathogenesis is dependent upon early replication within the draining lymph node, yet our understanding of how infection proceeds in this organ and its link to disease outcomes is incomplete (48). In this study, we sought to model MeV infection in primary human lymphoid tissue explants and comprehensively characterize the immunological events that occur following MeV infection. scRNA-Seq analysis of infected cells was made possible by the high infection rates achieved in this system coupled with a GFP-expressing pathogenic strain of MeV. Our study also contributes an analysis of the MeV-induced proteome within infected B cells. Our unbiased approaches suggest that MeV has a remarkably wide lymphoid tropism, as we found MeV transcripts in most of the 29 cell types identified within the lymphoid tissue explants. Our data verify previous findings of lymphocyte susceptibility and demonstrate a strong IFN signature associated with infection at both the RNA and protein levels.

Previous studies on MeV pathogenesis have identified a bias in infection toward B cells and away from T cells, with susceptibility differences driven by the expression of CD150 (18, 20, 27, 34, 40, 48, 49). Our data are consistent with this notion, as we found that B cells were the largest targets of infection, having heightened susceptibility and trending higher CD150 expression compared with T cells. Our analysis also extends these findings, revealing that while all B cells are highly susceptible to infection, accounting for the majority of infected cells, there were no observed differences in susceptibility based on B cell phenotype. Since CD150 expression was generally high among these B cell subsets, we interpret these findings to indicate that a baseline level of CD150 expression is sufficient to confer susceptibility, but differences beyond this threshold do not alter susceptibility. Of note, we found that germinal center B cells (GCBs) were by far the largest population of B cells found in this tissue, and thus, also comprised the greatest number of MeV-infected cells. These findings may suggest that immunological amnesia may extend beyond existing immunological memory to hamper future germinal center responses.

An interesting observation that we uncovered was that all cell types that we identified in the tonsil cultures were found to have some level of MeV transcripts. Several cell types had extremely low levels of MeV transcripts, including stromal and epithelial cells. While we took many measures to ensure that these were bona fide infected cells (such as dead cell removal, flow sorting for GFP, and a careful hashing strategy), we cannot eliminate the possibility that these cells are a byproduct of exosomes or ambient viral RNA co-encapsulated with the GFP^–^ populations rather than truly infected cells. Our findings in larger populations, such as subsets of CD4^+^ and CD8^+^ T cells, were recapitulated with our flow cytometry approach; however, future work should assess the possibility of MeV infection in rare tonsillar populations, such as stroma and epithelium.

Many early studies on MeV pathogenesis focused on infection of T cells within secondary lymphoid tissue (21, 34, 37, 43, 49, 50). Indeed, immunological amnesia was originally described as a T cell phenotype, where children who had previously tested positive for a hypersensitivity test to tuberculin antigen began to test negative following MeV infection (51). Subsequent work in thymus and macaques revealed that MeV preferentially infects and depletes memory T cells over naive T cell subsets, consistent with CD150 expression (32, 41). Our approach of assessing the relative susceptibility of both T cells broadly, as well as within individual subsets both confirmed and extended these findings. We found that antigen experience (CD45RA negativity) influenced susceptibility to infection, while CXCR5 expression, used here as a proxy for localization within the lymphoid explants, as well as CD150 expression, did not. Future work assessing the susceptibility of these antigen-experienced subsets should focus on directly testing if factors other than CD150 expression, such as spatial localization, promote susceptibility. Indeed, one parameter that may be interesting to evaluate would be the extent to which directed cell migration occurs within the tissue, and if infection influences immune cell trafficking.

Previous groups have shown that MeV does not induce a potent IFN response, as the viral V and C proteins can inhibit the induction of IFN (52–60). However, some groups have observed the opposite, where MeV induces potent IFN expression (46, 61, 62). In general, this discrepancy has been attributed to the presence of defective interfering (DI) RNAs, which can be enriched as a byproduct of in vitro replication (52, 59, 63, 64). While these would not be captured by our scRNA-Seq modality, we can conclude that the presence of viral V transcripts at day 8 was not sufficient to shut down the IFN response, whether induced by DI RNAs or viral replication. One hypothesis that might explain how MeV replicates in the presence of an IFN response would be that the IFN response is induced to the benefit of MeV, not the detriment. The idea that viruses may utilize IFN responses to promote infection has recently been demonstrated for influenza virus, whereby the virus utilizes the host ISG *IFIT2* to enhance the translational efficiency of viral RNAs (65). Alternatively, the addition of GFP into the viral genome may be indirectly involved as placement of GFP in the first transcriptional unit of our MeV-GFP may decrease the relative amounts of P-derived V and C proteins that antagonize type I IFN responses. MeV-C is known to reduce the production of DIs by enhancing the fidelity of the viral polymerase (66), with C-deficient MeV generating ~10-fold more DI RNAs than the parental virus (64). Future studies should assess the impact of this transcriptional shift on DI RNA production as well as the downstream ability to antagonize the type I IFN response.

One major limitation of our study is that we do not know the impact of MeV infection disease outcomes, such as immunological amnesia. Our results suggest that MeV infection of GCBs may impact the germinal center responses, an outcome that would amplify the impact of MeV on immunological amnesia. A second limitation of our study is that the transcriptomic and proteomic analyses were conducted in entirely different systems due to the heterogeneous nature of the lymphoid tissue explants. In the absence of single-cell proteomics, we limited our approach to a correlative analysis between the 2 methodologies and systems. Therefore, we have high confidence that these molecules are indeed a conserved response to MeV infection. A third limitation in our analysis is that we are unable to differentiate between cell death by MeV versus cell susceptibility to infection. While our data suggest that the broad susceptibility of T and B cells is positively associated with the expression of the entry receptor CD150, this does not exclude the possibility that some cell subpopulations have a greater capacity to survive while infected with MeV. Indeed, it has previously been established that MeV is capable of depleting CD150^+^ cells in tonsil explants (43). However, given the high similarity in the frequency of bystander cells and uninfected cells across all cell populations identified, we can presume that an enhanced frequency among GFP^+^ cells is indicative of enhanced susceptibility to infection. Understanding the impact of MeV on cell death and proliferation may prove critical to understanding the complete pathology of measles disease. Another limitation of our study is that we do not know the impact of the GFP produced by the MeV-GFP on the induction of the innate immune response in tonsil tissues and/or cell lines. The use of the GFP-expressing MeV enabled us to distinguish between infected and bystander cells in our culture system. This was unavoidable to sort and conduct scRNA-Seq for this study. However, a study utilizing this MeV-GFP showed that this strain is fully pathogenic in macaques, suggesting that the introduction of GFP, and its possible ISG induction, does not affect viral pathogenesis (67).

Our findings here represent a thorough analysis of the immunological events following MeV infection of human lymphoid tissue explants. The finding that MeV has a broad tropism within B cell, T cell, myeloid, and nonhematopoietic compartments may unlock new aspects of viral pathogenesis in humans. While we do not know the role of each of these cell types in the collective immunological response to infection, future studies should investigate how these cell types shape the progression of measles disease. Our findings also represent a model system for the testing of MeV antivirals, for which there are no current intervention strategies. One possibility would be that by targeting specific aspects of the induced IFN response, MeV pathogenesis could be ameliorated.

In total, we present a thorough kinetic examination of the process of MeV infection in human lymphoid explants, verifying previous groups’ findings and broadening our understanding of the key players in MeV infection within its natural target organ architecture. Further, our integrated transcriptional and proteomic approach in this model establishes tonsil explants as a potentially unique platform for the identification of host factors important for MeV replication and screening of targeted antivirals. Future work in this model should focus on understanding how MeV replicates in the face of this potently induced IFN response to identify junctions at which viral replication can be inhibited.

## Methods

### Sex as a biological variable.

Our study received human tonsil tissue from both male and female donors. We did not observe any clear sex difference in MeV replication, so these data were analyzed together.

### Cells and plasmids.

Vero-hCD150 cells were provided by Yusuke Yanagi at Kyushu University in Fukuoka, Japan, and maintained in DMEM with 10% FBS (Biowest). Raji-DCSIGNR cells were gifted by Ted Pierson (NIH Vaccine Research Center, Bethesda, Maryland, USA) and cultured in RPMI with 10% FBS (68). The genome coding plasmid for MeV, p(+) MV323-AcGFP, was gifted from Makoto Takeda (University of Tokyo, Tokyo, Japan) (47). The MeV genome sequence was transferred into a pEMC vector, adding an optimal T7 promoter, a hammerhead ribozyme, and an eGFP transcriptional unit at the 3′ end of the genome (pEMC-IC323-eGFP) as previously described (47).

### MeV rescue and amplification.

MeV (IC323-eGFP) rescue was performed in BSR-T7 cells, seeded in 6-well format. Upon confluence, pEMC-IC323eGFP (5 μg), T7-MeV-N (1.2 μg), T7-MeV-P (1.2 μg), T7-MeV-L (0.4 μg), a plasmid encoding a codon-optimized T7 polymerase (3 μg), PLUS reagent (5.8 μL, Invitrogen), and Lipofectamine LTX (9.3 μL, Invitrogen) were combined in Opti-MEM (200 μL; Invitrogen). After a 30-minute incubation at room temperature (RT), the transfection mixture was added dropwise onto cells and incubated for 24 hours at 37°C. Following that, rescued virus was amplified once on Vero-hCD150 cells for 72 hours to generate a P1 virus, in infection media (made in DMEM + 2% FBS). This virus was then titered (see plaque assay method below) and used at an MOI = 0.01 on Vero-hCD150 cells to generate a P2 virus (amplified as above). Supernatants were collected, clarified of cell debris, ultracentrifuged through a 20% sucrose gradient at 76,124*g* for 3 hours, reconstituted in fresh infection media, and frozen at –80°C.

### MeV quantification by plaque assay.

Vero-hCD150 were plated in 12-well format until approximately 90%–95% confluent. Then 10-fold dilutions of samples (made in DMEM + 2% FBS) were applied to these monolayers in a total volume of 250 μL, and infections were allowed to incubate for 2 hours at 37°C. Viral inoculum was replaced with 500 μL/well of methylcellulose (in DMEM + 2% FBS + 7.5% NaHCO_3_). At 72 hours, wells were imaged for GFP^+^ plaques on the Celigo S platform.

### Processing and infection of human lymphoid tissue.

Human tonsils from routine tonsillectomies performed at the Mount Sinai Hospital and the New York Eye and Ear Infirmary of Mount Sinai were collected under IRB-approved protocols within a few hours after surgery. Tonsils were cut into 2 mm^3^ blocks, and 9 tissue blocks per well were placed on top of collagen gel foams (Cardinal Health) in a 6-well plate as previously described, utilizing 3 wells per condition (a total of 27 blocks per experimental sample) (43). In all experiments, triplicate wells were harvested as a single sample to reduce variability (44). After overnight incubation, individual tissue blocks were individually inoculated with 5 μL containing 1,666 PFU MeV-GFP (for a final concentration of 5,000 PFU/mL) or left uninfected. Medium was collected and replaced at days 3, 6, and 8 after infection. Tonsil donors consisted of 3 male and 8 female donors. The median age of donors was 23 years old, with a range of 4 to 54 years old. The reasons for tonsillectomy included sleep apnea, breathing disorders, and chronic tonsillitis.

### Visualization of MeV-infected cells in tonsillar explants by in situ hybridization.

Tissues were fixed in 10% neutral buffered formalin and paraffin-embedded. In situ hybridization using RNAscope (ACDBio) was performed on 5 μm sections to detect RNA encoding GFP. Deparaffinization was performed by baking slides at 55°C for 20 minutes. Slides were washed twice with xylene, then twice in 100% ethanol, and were dried for 5 minutes at 60°C. Slides were then incubated with hydrogen peroxide for 10 minutes at RT and were subsequently washed in deionized H_2_O. Slides were placed in Target Retrieval solution at 100°C for 15 minutes, washed with water, and transferred into 100% ethanol for 3 minutes, before drying. Sections were treated with RNAscope Protease Plus, and fluorescence in situ hybridization was subsequently performed according to the manufacturer’s protocol (ACDBio 323110) with RNAscope Probe EGFP (ACDBio 400281; binds eGFP RNA) as previously described (69). Slides were then mounted with Vectashield hard-set mounting medium with DAPI (Vector Laboratories) and analyzed using an AxioImager Z2 microscope (Zeiss) and Zen 2012 software (Zeiss).

### Generating single-cell suspensions from tonsil histocultures.

Single-cell suspensions were generated by dissociating tissue (merged from the 3 technical triplicate wells) using Collagenase IV (Worthington Biochemical) incubated for 30 minutes at 37°C with gentle shaking as previously described (44). Samples were homogenized with mortar and pestle before filtration over a 100 μm cell filter and washed once with cold PBS before downstream application.

### scRNA-Seq.

Samples for scRNA-Seq were pooled for multiplex processing and analysis with a cell hashing antibody strategy (70). Hash antibodies were generated by conjugating Integrated DNA Technologies synthesized oligos (barcode sequences from 10x Genomics Chromium index SI-GA-F11; HBC21-29 for hash 1–8) to antibodies utilizing Thunder-Link PLUS oligo Antibody Conjugation Kit. Single-cell suspensions were generated from 1 donor-matched infected and uninfected culture at day 8, and dead cells were depleted from samples using the EasySep Dead Cell Removal (Annexin V) Kit (STEMCELL Technologies, 17899). Cells were blocked with Human TruStain FcX (BioLegend, 422302). Cells from the uninfected tonsil were split into 4 hashing groups (hash 1–4), and cells from the infected tonsil were split into 2 infected hashing groups (hash 5, 6) and 2 bystander hashing groups (hash 7, 8). Samples were stained with corresponding hashing antibodies (a pool of anti-CD298 and anti-B2M conjugated to barcoded synthesized oligos as described above; clones LNH-94 and 2M2, respectively) for 30 minutes at 4°C and washed 3 times in FACS buffer (PBS + 1 mM EDTA + 2% BSA). Cell suspensions were filtered over a 70 μm filter (Falcon, Corning), stained with propidium iodide for viability, and sorted as live/GFP^–^ cells from the uninfected condition, live/GFP^+^ cells from the infected condition, and live/GFP^–^ cells from the bystander condition on a BD FACSAria III. Sorted cells were counted, and 10,000 uninfected cells, 5,000 infected cells, and 5,000 bystander cells were pooled and processed for scRNA-Seq on the 10x Genomics Chromium platform, utilizing the 10x 3′ v3 kit. An scRNA-Seq library was generated as per the manufacturer’s protocol and sequenced on an Illumina NextSeq500 instrument. A corresponding library of barcoded hash antibody oligonucleotides was indexed with a standard Illumina D701 index and sequenced as above.

### Processing of scRNA-Seq data.

Raw sequencing data output (BCL files) was converted to FASTQ files with CellRanger mkfastq v3.0.2 (10x Genomics). Per-cell gene count and hashtag antibody count matrices were generated with CellRanger count v3.0.2 (10x Genomics), using a human genome reference (GRCh38, Ensembl v96 transcript annotations) appended with the MeV-eGFP reference and corresponding transcript annotations (MeV-IC323-eGFP, GenBank MW401770). Data were read into the R statistical framework (v4.0.3) for additional analysis with Seurat (71, 72) (v4.0.1). Hashtag antibody data were center log ratio–normalized by feature, and individual samples were demultiplexed with the Seurat HTODemux function with the positive.quantile parameter set to 0.99.

### Quality control and filtering of scRNA-Seq.

Data exploration and HTODemux classifications were used to set quality control thresholds on per-cell transcript UMI counts, detected gene counts, and the percentage of detected mitochondrial transcripts. Cells with fewer than 2,500 transcript UMIs, fewer than 800 detected genes, and greater than 15% mitochondrial transcripts were excluded from downstream analyses. After filtering, these data included 5,737 cells in the uninfected group, 2,736 cells in the infected group, and 2,944 in the bystander group.

### scRNA-Seq data analysis.

Datasets were normalized with SCTransform (73), with the per-cell mitochondrial transcript percentage included as a regression variable. Data from all groups were integrated in Seurat using 3,000 anchor features; MeV genes were excluded from all integration and clustering steps to avoid group-specific artifacts. Dimensionality reduction was performed by principal component analysis on integrated data, and the first 20 components were selected for graph-based clustering by smart local moving algorithm (74) at a resolution of 1.4 (determined by clustering tree assessment, ref. 75).

General cell types were annotated by SingleR (76) from human immune cell reference data (77). Clusters were assigned to one of each major cell group: T/NK, B, plasma, myeloid, stromal, and epithelial. Those major cell groups with multiple component clusters were subset and reanalyzed (normalization, principal components analysis dimensionality reduction, and clustering at clustree-determined optimal resolution) for further annotation. For each major cell group subset analysis, “marker genes” distinguishing component clusters were identified with the FindAllMarkers (on the uninfected group) or FindConservedMarkers (on all groups) functions. Intergroup differential gene expression analysis was performed with edgeR (78, 79) (v3.32.1), including modifications of scRNA-Seq data (80). The edgeR linear model incorporated factors for cellular gene detection rate (to account for scRNA-Seq “dropout”) and experimental group and included only those genes detected in at least 20% of cells in any contrast condition. Statistical thresholds were set at Benjamini-Hochberg–adjusted *P* value less than 0.0001 and absolute log fold-change greater than 1.58 for differential expression.

### Immunophenotyping by flow cytometry.

Cells were stained with the Zombie Red fixable viability kit (BioLegend, 423109) for 10 minutes at RT, washed once with FACS buffer, and then blocked with Human TruStain FcX (BioLegend, 422302). Samples were incubated for 30 minutes on ice with a cocktail of antibodies against (B Cell Panel) CD150 (BioLegend; clone: A127d4; PE), CD38 (eBioscience; clone: HB7; PE-Cy7), CD27 (BioLegend; clone: O323; APC), CD45 (BD Horizon; clone: HI30; BV605), CD19 (BioLegend; clone: HIB19; BV750), and CD3 (BioLegend; clone: OKT3; BV785) and (T Cell Panel) CD4 (eBioscience; clone: OKT4; PerCP-Cy5.5), CD150 (BioLegend; clone: A127d4; PE), CD45RA (BioLegend; clone: HI100; Alexa Fluor 700), CXCR5 (BioLegend; clone: J252D4; BV421), CD8 (BioLegend; clone: RPA-T8; BV570), CD45 (BD Horizon; clone: HI30; BV605), CD19 (BioLegend; clone: HIB19; BV750), and CD3 (BioLegend; clone: OKT3; BV785). The antibody cocktail was supplemented with Brilliant Stain buffer (BD Horizon, 563794). All antibodies were used at a concentration of 1 μg/mL, except for CD27 (4 μg/mL). Cells were washed 3 times with FACS buffer before fixation with Cytofix (BD Biosciences; 554655). Single-color controls were generated on UltraComp eBeads (Invitrogen, 01-2222-42), except for GFP and Live/Dead controls, which were generated using cells. All samples were analyzed on an Aurora Cytek, and unmixed samples were analyzed in FlowJo v10.8.1.

### qRT-PCR.

Resuspended single–tonsil cell suspensions were placed in 1 mL of TRIzol (Invitrogen), and RNA was isolated using Direct-zol RNA MiniPrep Plus kit (Zymo). A total of 1 μg of RNA was reverse-transcribed with random hexamer primers (Applied Biosystems). A total of 1 μL of cDNA was utilized per reaction, and primer/probes for *IFIT1* (HS03027069_S1), *IFIT3* (HS01922752_S1), and *MX1* (Hs00895608_m) were utilized to amplify ISG transcripts. Fold-change in induction was calculated using the ΔΔCT method by normalizing expression to *GAPDH* expression (NC_000012.11).

### Sample preparation for mass spectrometry.

Uninfected or MeV-infected Raji-DCSIGNR cells were lysed in 8 M urea lysis buffer (with 100 mM ammonium bicarbonate, 150 mM NaCl, and 1× protease/phosphatase inhibitor cocktail HALT from Thermo Fisher Scientific). Lysates were sonicated, and protein concentrations were quantified by micro-BCA assay (Thermo Fisher Scientific). A total of 50 μg of protein for each sample was treated with Tris-(2-carboxyethyl)phosphine at a 4 mM final concentration and incubated for 30 minutes at RT. Iodoacetamide (IAA) was added to a 10 mM final concentration, and samples were incubated for 30 minutes at RT. Free IAA was quenched with the addition of dithiothreitol at a 10 mM final concentration for 30 minutes. Samples were diluted with 5 sample volumes of 100 mM ammonium bicarbonate. Lysates were next digested with Trypsin Gold (Promega) at a 1:100 (enzyme/protein) ratio, and lysates were rotated for 16 hours at RT. Trypsin activity was quenched by adding 10% v/v trifluoroacetic acid (TFA) to a final concentration of 0.1%. Samples were desalted on BioPure SPN MIDI C18 Spin columns. Samples were eluted from these columns with 200 μL 40% acetonitrile (ACN)/0.1% TFA, dried by vacuum centrifugation, and stored at –80°C.

### Protein abundance mass spectrometry.

Samples were analyzed on an Orbitrap Eclipse mass spectrometry (MS) system (Thermo Fisher Scientific) equipped with an Easy nLC 1200 ultra-high pressure liquid chromatography system (Thermo Fisher Scientific) interfaced via a Nanospray Flex nanoelectrospray source. Immediately before spectrometry, lyophilized samples were resuspended in 0.1% formic acid (FA). Samples were injected on a C18 reverse phase column (30 cm × 75 μm, inner diameter) packed with ReprosilPur 1.9 μm particles). Mobile phase A consisted of 0.1% FA, and mobile phase B consisted of 0.1% FA/80% ACN. Peptides were separated by an organic gradient from 5% to 35% mobile phase B over 120 minutes followed by an increase to 100% B over 10 minutes at a flow rate of 300 nL/min. Analytical columns were equilibrated with 3 μL of mobile phase A. To build a spectral library, samples from each set of biological replicates were pooled and acquired in a data-dependent manner. Data-dependent analysis was performed by acquiring a full scan over an *m/z* range of 375–1,025 in the Orbitrap at 120,000 resolution (at 200 *m/z*) with a normalized automatic gain control (AGC) target of 100%, a radio frequency (RF) lens setting of 30%, and an instrument-controlled ion injection time. Dynamic exclusion was set to 30 seconds, with a 10 parts per million exclusion width setting. Peptides with charge states 2–6 were selected for MS/MS interrogation using higher energy collisional dissociation (HCD) with a normalized HCD collision energy of 28%, with 3 seconds of MS/MS scans per cycle. Data-independent analysis (DIA) was performed on all individual samples. An MS scan was performed at 60,000 resolution (*m/z* 200) over a scan range of *m/z* 390–1,010, an instrument-controlled AGC target, an RF lens setting of 30%, and an instrument-controlled maximum injection time, followed by DIA scans using *m/z* 8 isolation windows over *m/z* 400–1,000 at a normalized HCD collision energy of 28%.

### MS data analysis.

Peptides/proteins were first identified with Spectronaut (81). False discovery rates (FDRs) were estimated using a decoy database strategy. All data were filtered to achieve an FDR of 0.01 for peptide-spectrum matches, peptide identifications, and protein identifications. Search parameters included a fixed modification for carbamidomethyl cysteine and variable modifications for N-terminal protein acetylation and methionine oxidation. All other search parameters were defaults for the respective algorithms. Analysis of protein expression utilized the MSstats statistical package in R. Output data from Spectronaut was annotated based on a publicly available *Homo sapiens* proteome (Proteome ID UP000005640) and the reference sequence for IC323-eGFP (GenBank MW401770.1). Technical and biological replicates were integrated to estimate log_2_ fold-changes, *P* values, and adjusted *P* values. All data were normalized by equalizing median intensities, the summary method was Tukey’s median polish, and the maximum quantile for deciding censored missing values was 0.999. Significantly dysregulated proteins were defined as those that had a fold-change > 2 or < –2, with a *P* value of less than 0.05. The mass indices for the most significantly dysregulated proteins were transformed with the quantile function in R and then visualized using the pheatmap package.

### GO analysis.

GO enrichment analysis was performed using a hypergeometric test with the dhyper function in R. GO annotations were downloaded from UniProt and GO definitions from the Gene Ontology Resource on February 18, 2021. The test sets comprised proteins significantly increasing or decreasing (i.e., |log_2_fold-change| > 1 and adjusted *P* < 0.05, excluding infinity values) in each comparison of interest, and the background set was all proteins quantified in the comparison of interest. Enrichment tests were performed for any GO term that had at least 2 overlapping proteins in the test set. Proteins identified by peptides that were not unique to a single protein sequence were excluded from this analysis.

### Western blot analysis of Raji cell lysates.

Raji cells were infected with MeV at an MOI = 0.1 for 72 hours and compared with uninfected controls (*n* = 3). Cells were lysed as described above, and 10 μg samples of whole-cell lysate was mixed 1:1 with Laemmli buffer (containing β-mercaptoethanol) and heated at 95°C for 10 minutes. Samples were then electrophoresed on a 4%–20% gradient SDS-PAGE gel (Bio-Rad) and transferred onto a methanol-activated PVDF membrane (Bio-Rad). The membrane was blocked with 5% milk in PBS with 0.1% Tween 20 for 1 hour. The following antibody staining protocols were run sequentially: 1) anti-IFIT3 (Thermo Fisher Scientific; clone: OTI1G1; 1:1,000) developed with goat anti-mouse IgG-HRP (catalog: G-21040; 1:10,000), 2) anti-ISG15 (clone: 7H29L24; 1:5,000) developed with goat anti-rabbit IgG-HRP antibody (Thermo Fisher Scientific; catalog: 65-6120; 1:10,000), and 3) anti–β-Actin (Thermo Fisher Scientific; clone: 15G5A11/E2; 1:1,000) and anti-GFP (Thermo Fisher Scientific; clone: GF28R; 1:1,000) simultaneously, developed with anti-mouse Alexa Fluor 647 antibody (Thermo Fisher Scientific; clone A-21235; 1:2,000). HRP signals were detected between each incubation with SuperSignal West Pico PLUS reagent (Thermo Fisher Scientific; 1:1 luminol/enhancer), and images were acquired on a Chemidoc MP. Western images were merged for presentation in Fiji.

### Correlation analysis of RNA and protein response to infection.

Data from scRNA-Seq and MS were further processed in RStudio to correlate RNA and protein levels. All B cells from the scRNA-Seq dataset were rebulked, and total read counts in this new “B cell” cluster were calculated, normalized to counts/kb million, and log_2_-transformed. To evaluate the fold-change between mock-treated and MeV-infected samples, the log_2_ values from the mock condition were subtracted from the MeV condition. A correlation scatterplot was created using ggplot, and protein labels were added only if the log_2_ fold-change values were greater than 2 in both RNA and protein.

### Bulk RNA sequencing of infected Raji cells.

Raji-DCSIGNR cells were infected with MeV as during the preparation of MS samples above. Cells were pelleted and resuspended in 500 μL of TRIzol. RNA was extracted using the Direct-zol RNA Miniprep kit (Zymo Research), and frozen RNA was shipped to Azenta Life Sciences for library preparation and sequencing. ERCC RNA Spike-in Max kit was added to normalize total RNA prior to library preparation following the manufacturer’s protocol (catalog 4456740). RNA-sequencing libraries were prepared using the New England Biolabs NEBNext Ultra II RNA Library Prep Kit for Illumina. Libraries were quantified with Agilent TapeStation, Qubit 2.0, and by quantitative PCR prior to sequencing on an Illumina NovaSeq X Plus 25B. Samples were sequenced using a standard 2 × 150 bp paired-end configuration. Raw sequence data were converted into FASTQ files and demultiplexed using Illumina’s bcl2fastq 2.2.0 software. The quality of sequencing was assessed with FastQC, and sequencing reads were aligned to indexed reference genomes using the STAR aligner. Expression matrices were calculated using featureCounts, and data were exported into R for data analysis and visualization. Transcripts where fewer than 10 transcripts were collected across all samples were excluded from further analysis. Data normalization and differential gene expression analysis were conducted using the DESEQ2 package (version 1.42.1).

### Statistics.

For scRNA-Seq and mass spectrometry analysis, statistical analysis methodology is detailed in the above Methods subsections. For all comparisons of infection susceptibility over time, significance was determined by 2-way ANOVA using the Geisser-Greenhouse correction with Tukey’s multiple-comparison test. For all comparisons of CD150 expression among multiple (>2) groups, significance was determined by 1-way ANOVA using Friedman’s test with Dunnett’s multiple-comparison test. For pairwise comparisons, a nonparametric 2-tailed paired *t* test was utilized (Wilcoxon’s matched pairs signed rank test). For experiments with 3 replicates, the median with the 95% confidence intervals is shown instead of *P* value. *P* < 0.05 was considered statistically significant.

### Study approval.

All tonsil tissues were obtained with written informed consent under IRB 16-01425-CR002 at the New York Eye and Ear Infirmary of Mount Sinai or the Mount Sinai Hospital, New York, New York, USA, under IRB HS12-0045.

### Data availability.

Large datasets will be made available on NCBI GEO and SRA: bulk sequencing (accession GSE272426) and scRNA-Seq (accession GSE272481). The mass spectrometry proteomics data have been deposited to the ProteomeXchange Consortium via the PRIDE (82) partner repository with the dataset identifier PXD054861.

Raw data values for data shown in this manuscript can be accessed in the Supporting Data Values XLS file. Information about human participants is limited by the IRB; however, anonymized information will be made available upon request to the corresponding author where possible.

## Author contributions

JAA, BRR, BL, and JKL conceptualized the project. JAA, ARP, SH, APK, PAT, SI, JC, HU, BT, JRJ, BRR, BL, and JKL contributed to the work intellectually. JAA, ARP, SH, ASM, PAT, NI, and SK conducted the experiments. SI generated the virus used in this study. BT conducted tonsillectomies included in this work. JAA, ARP, SH, EJD, and BRR conducted data analysis. JAA, ARP, and JKL prepared the manuscript. All authors edited the manuscript. The order of the co–first authors was determined based on contributions to the conceptualization and execution of this work.

## Supplementary Material

Supplemental data

Unedited blot and gel images

Supporting data values

## Version 1 09/10/2024

Electronic publication
