# Supplementary material for: Immunological landscape of human lymphoid explants during measles virus infection
Source: JCI Insight. 2024 Jul 25;9(17):e172261. doi: 10.1172/jci.insight.172261 (PMC11385098; doi:10.1172/jci.insight.172261)
Supplement: Supplemental data [file jciinsight-9-172261-s119.pdf]

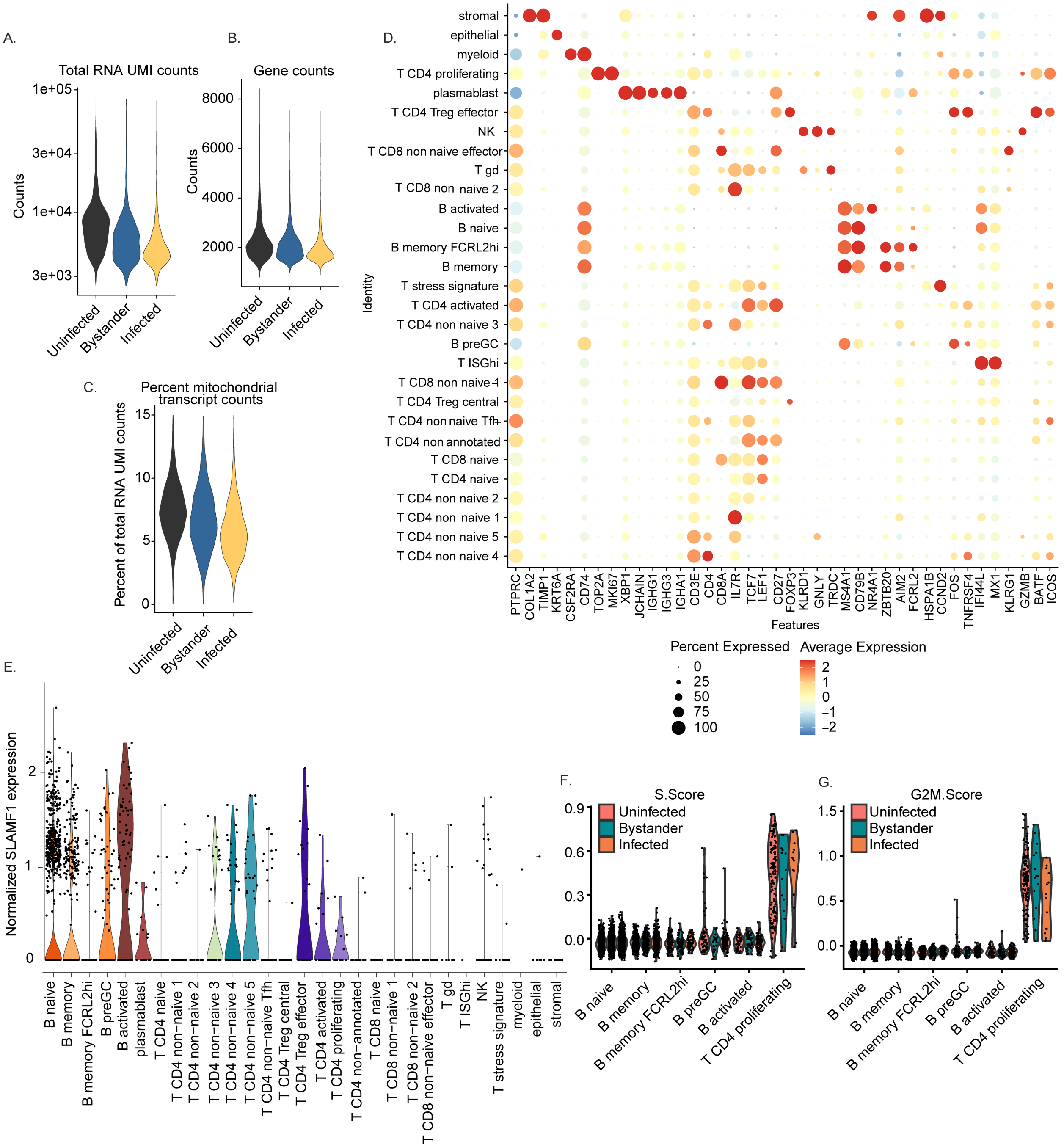

Figure S1 (related to Figure 2): Cluster annotation strategy for scRNA-Seq. Total counts of unique molecular identifiers (UMI; **A**), individual genes **(B)**, and mitochondrial transcripts **(C)** were quantified for quality control and filtering. Dot plot showing the expression of cluster-defining features across each identified cell cluster. Average expression (Z scaled) is shown for each feature by color, while the percentage of cells in each cluster expressing that feature is shown by the size of the dot **(D)**. Normalized SLAMF1 expression across all cell types was determined **(E)**. Pathway scores for S phase **(F)** and G2M **(G)** were calculated and compared across B cell subsets and proliferating CD4 T cells.

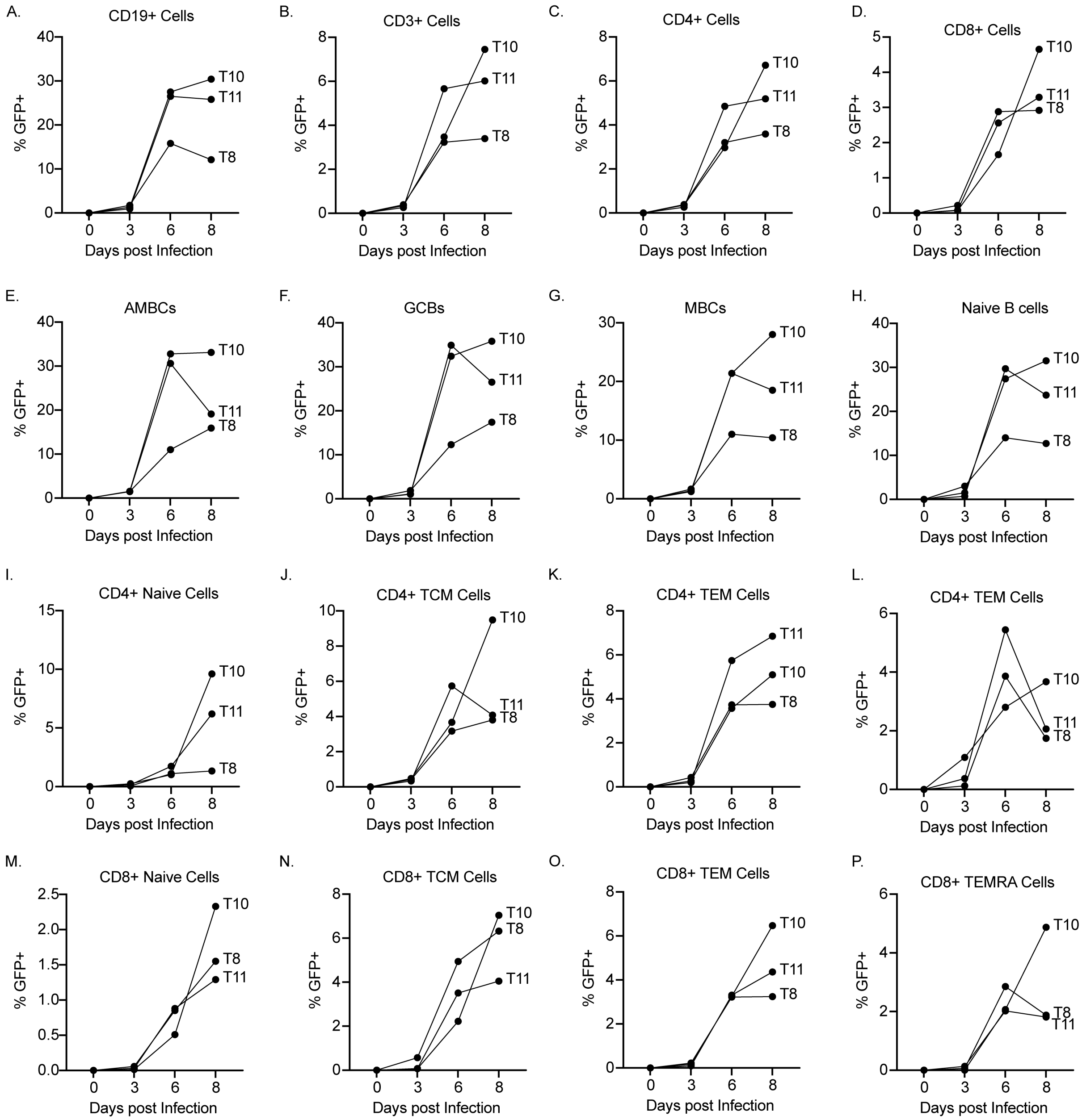

Figure S2 (Related to Figure 3): Quantification of infection kinetics in different cell populations. Secondary analysis of donors from Figure 3. The percentage of GFP^+ cells within each cell type identified in the explants is quantified for B and T cells **(A-B)**, $CD4^+$ and $CD8^+$ cells **(C-D)**, B cell subsets **(E-H)**, $CD4^+$ T cell subsets **(I-L)** and $CD8^+$ T cell subsets **(M-P)**. Each dot/line represents a single donor.

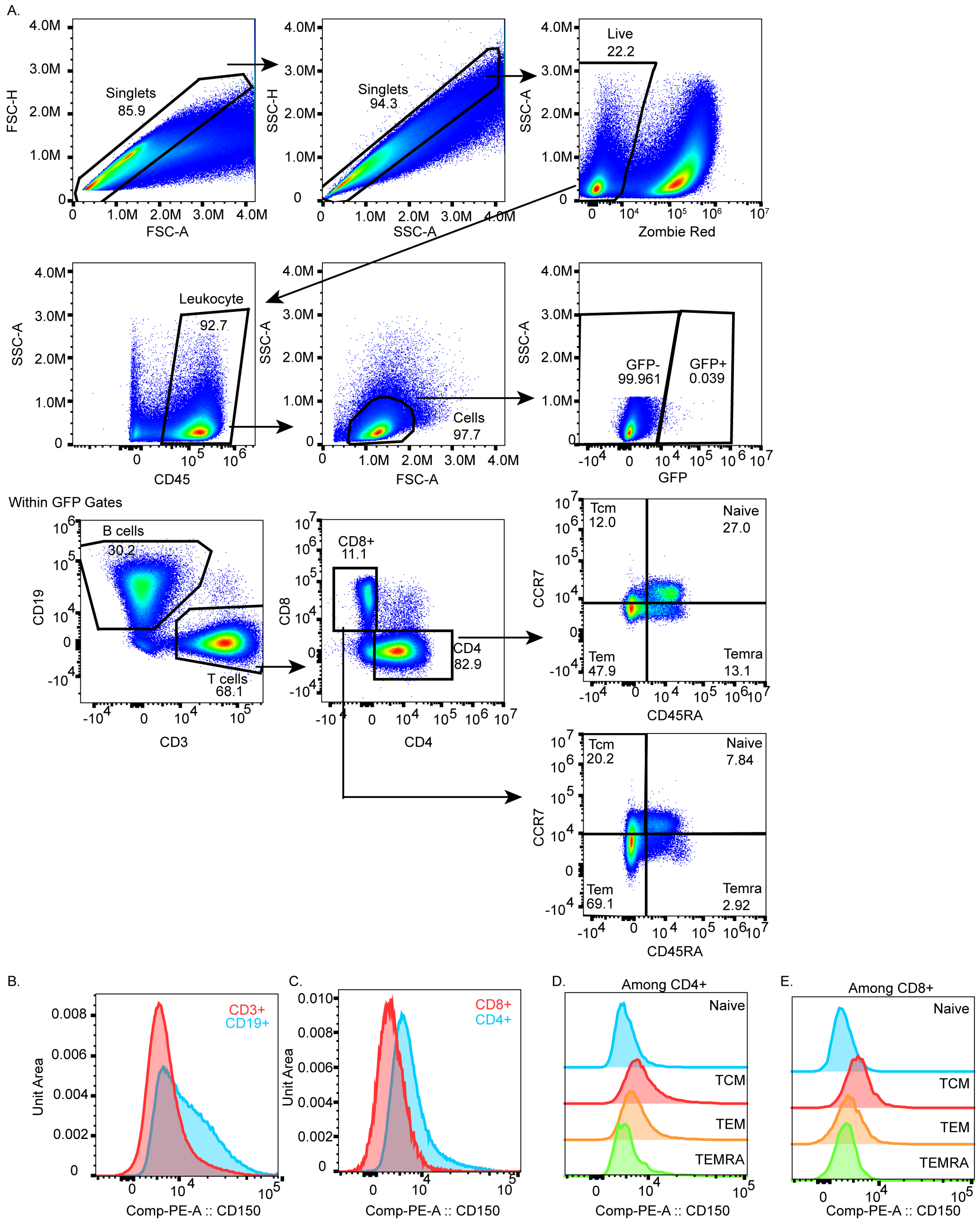

Figure S3 (Related to Figure 3). Gating schemata for flow cytometry-based immunophenotyping experiments. Representative flow plots are shown for one uninfected donor. Single cells are selected, followed by dead cell exclusion, gating upon CD45$^+$ cells, cell gating, and then binning cells based on GFP status **(A)**. Within GFP$^+$ or GFP- gates, cells are further phenotyped based on CD3/CD19 (B vs T cells), CD4 vs CD8 lineages within the CD3$^+$ gate, and then memory subsets within these lineages. Histograms demonstrating CD150 expression are compared between CD3$^+$ and CD19$^+$ cells **(B)**, between CD4$^+$ and CD8$^+$ cells **(C)**, or between CD4$^+$ **(D)** or CD8$^+$ **(E)** memory subsets for one representative donor in the day 6 uninfected condition.

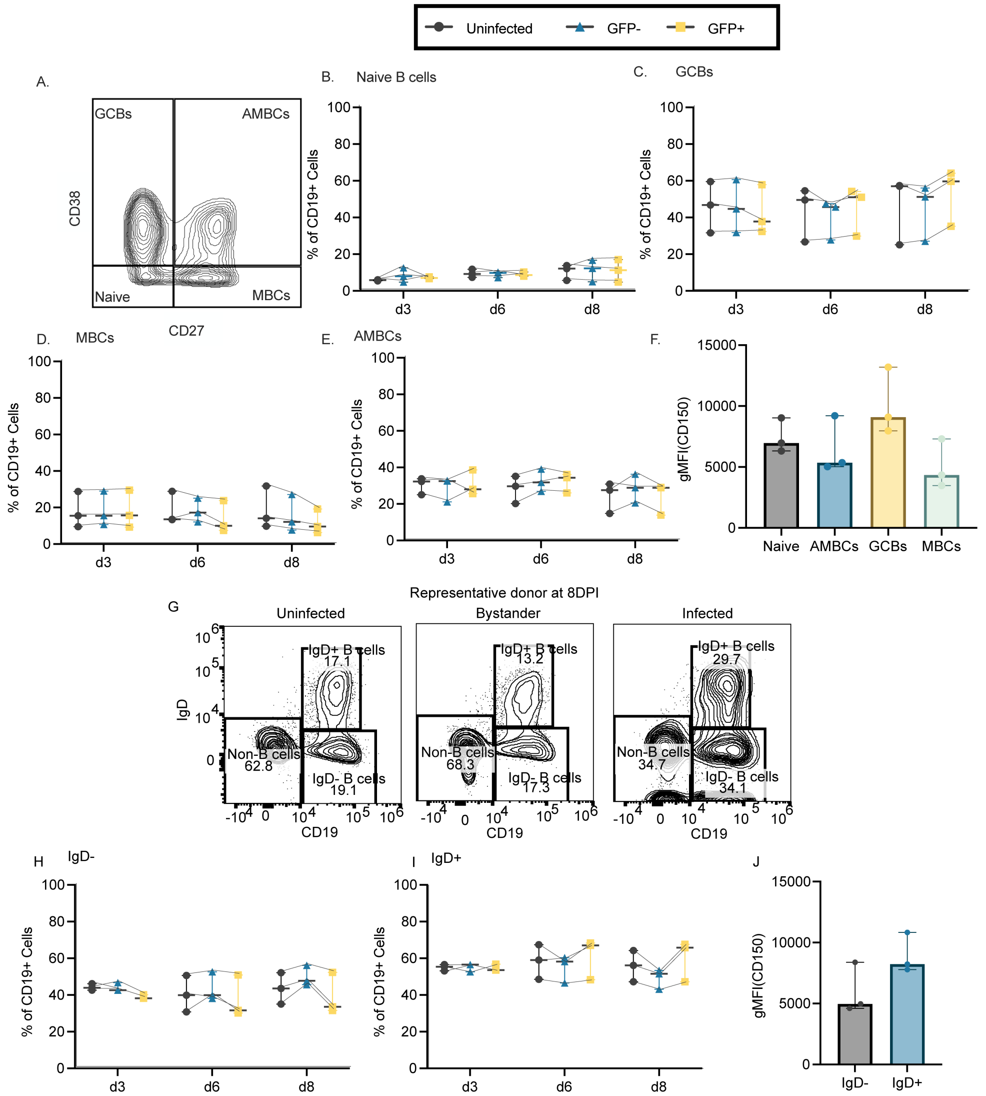

Figure S4 (related to Figure 3): MeV infects B cell subsets proportionally. Donors (n=3) from Figure 3 were immunophenotyped to identify B cell subsets using CD38 and CD27 within the $CD19^{+}$ cells, with the gating strategy utilized shown in **(A)**. Naive ($CD27^{-}CD38^{-}$; **B**), germinal center ($CD27^{-}CD38^{-}$; **C**), memory ($CD27^{+}CD38^{-}$; **D**), and activated memory ($CD27^{+}CD38^{+}$; **E**) B cells were quantified by comparing their frequency among both uninfected and GFP^{+} cells over time. CD150 expression was calculated for each population among uninfected cells at 6 days post-infection and the mean fluorescent intensity (MFI) of CD150 expression is shown **(F)**. Representative flow plots are shown demonstrating the enrichment of IgD^{+} B cells among all infected cells at 8DPI **(G)**. Susceptibility to infection was assessed by quantifying the frequency of IgD- **(H)** or IgD^{+} **(I)** cells among uninfected, bystander, or infected cells. CD150 expression on IGD- and IgD^{+} populations at day 6 are shown in **(J)**. For all immunophenotyping panels**,** significance was determined by two-way ANOVA using the Geisser-Greenhouse correction with Tukey's multiple comparison test. For panel **(F)** significance was determined by one-way ANOVA using Friedman's test with Dunnett's multiple comparison test. Significance in **(J)** was determined using the Wilcoxon matched-pairs signed rank test. For all plots, the median with the 95% confidence interval is shown.

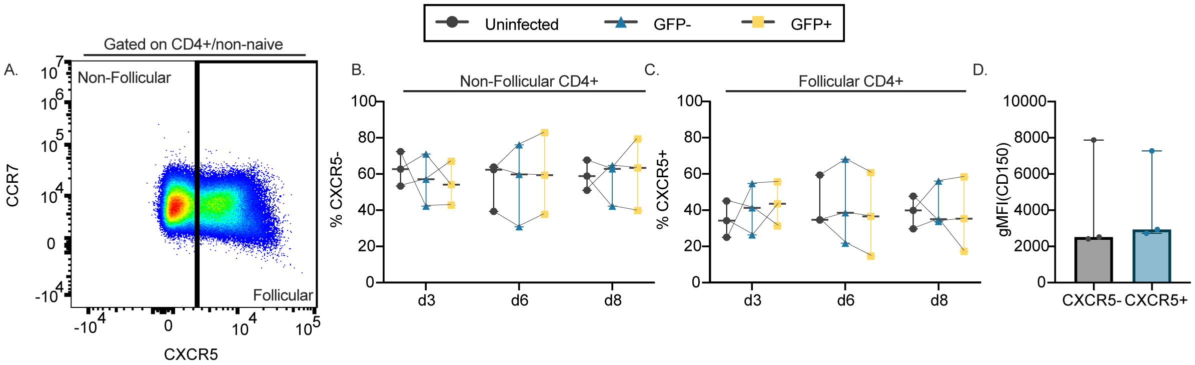

Figure S5 (related to Figure 3): CXCR5 status has no impact on the susceptibility of CD4$^+$ cells. Non-Naïve CD4$^+$ cells (n=3) were subset based on CXCR5 status as shown in (**A**). Shown are the frequency of non-follicular (CXCR5$^-$; **B**) and follicular (CXCR5$^+$; **C**) cells among uninfected, GFP$^-$ (bystander), or GFP$^+$ non-naïve CD4$^+$ cells. CD150 expression was compared between non-follicular and follicular cells (**D**). Significance for **B-C** was determined by two-way ANOVA using the Geisser-Greenhouse correction with Tukey's multiple comparison test, and CD150 expression significance was determined by the Wilcoxon matched-pairs signed rank test (**D)**. For all plots, the median with the 95% confidence interval are shown.

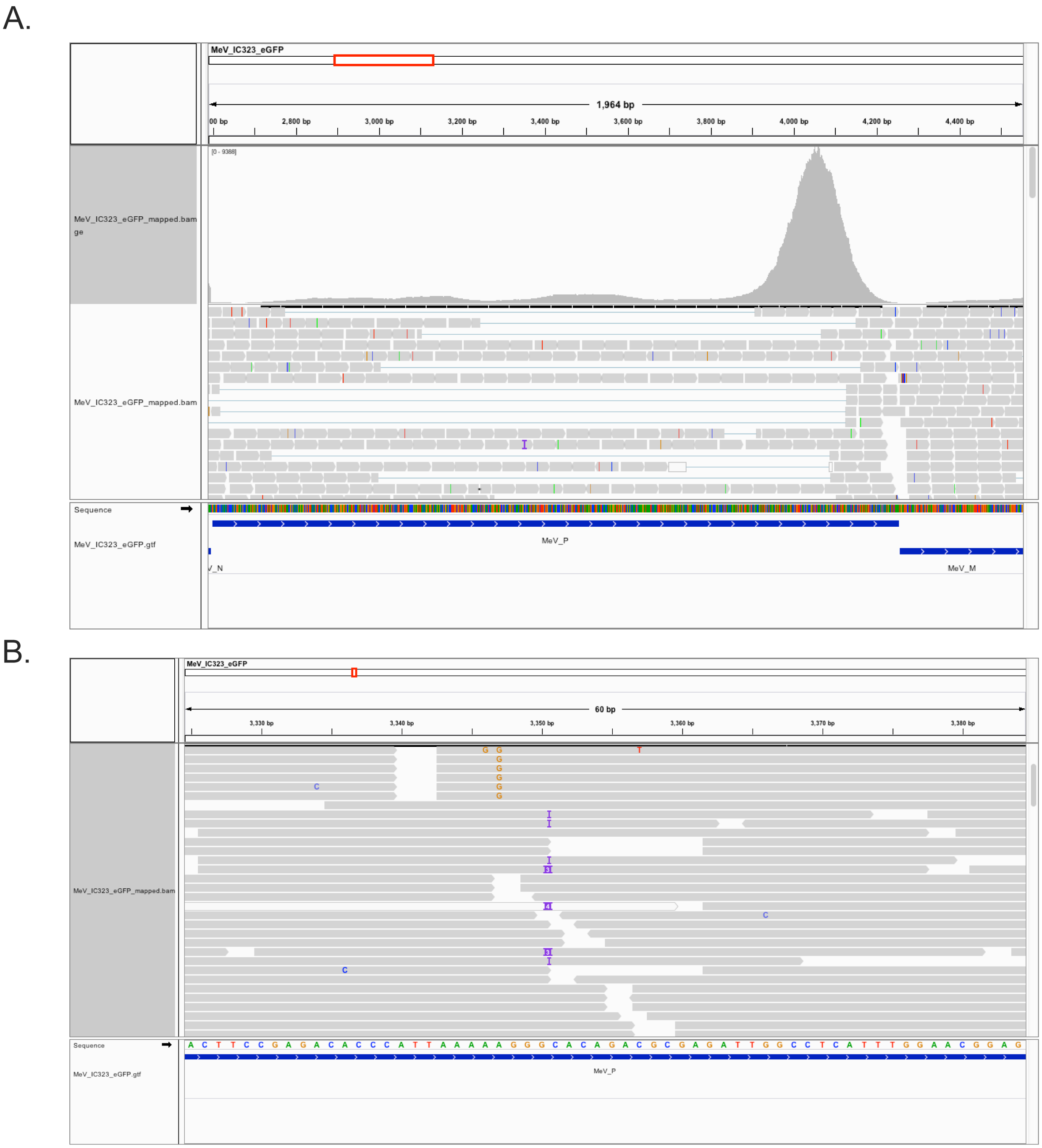

Figure S6 (related to Figures 2 and 4): Detection of P-editing by scRNA-Seq. (A) Read coverage for all libraries along the length of the MeV P transcript. The red box denotes the region of the MeV linear genome that is shown in the histogram box, and the boxes under the histogram are a selection of reads that map to these positions. Gray bars indicate that the read matches the reference sequence, with colored letters representing mismatches to the reference. **(B)** Zoomed-in coverage of the p-edited region, showing representative reads that map to this region of the gene in gray. "I" represents indel mappings, which may indicate P-edited transcripts. There were 235 total reads covering the putative edit site.

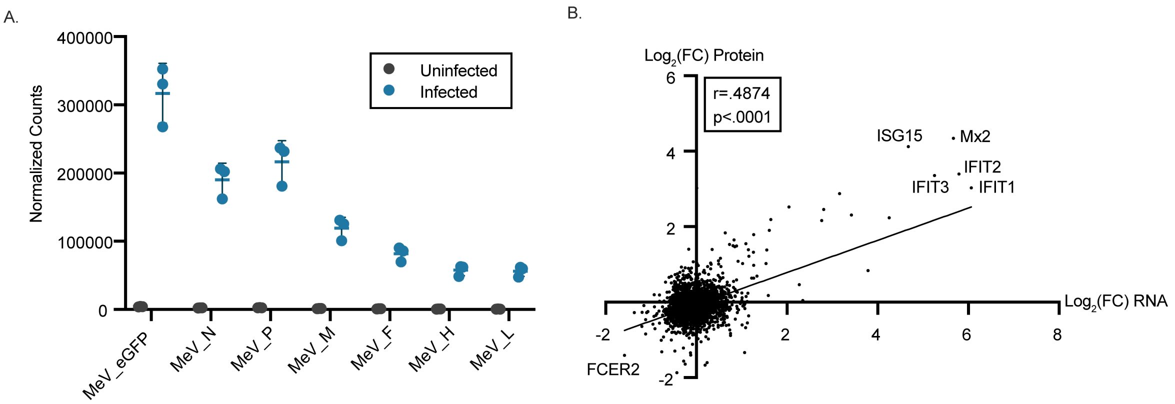

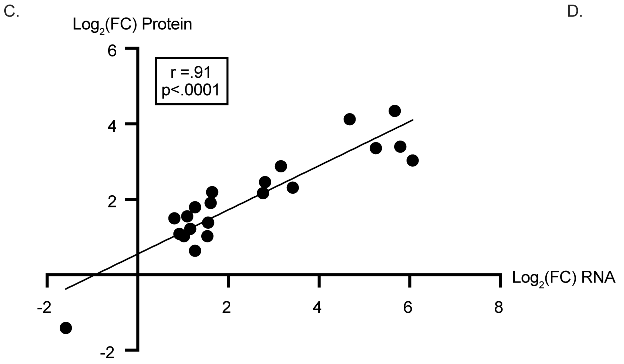

D.

Conserved Host Rersponse (RAJI)

	Log_2FC(RNA)	Log_2FC(Protein)
FCER2	-1.588285800	-1.408321500
APOL3	0.808886240	1.493501530
SP100	0.918920840	1.083946100
PNPT1	1.016999930	1.026074210
NMI	1.091598070	1.546900570
PML	1.158179410	1.219792500
CD38	1.264930940	0.633885620
DTX3L	1.265487070	1.788830760
N4BP1	1.538674880	1.020341890
PARP9	1.551765320	1.383604620
ISG20	1.606015940	1.906262910
STAT2	1.639357310	2.189254300
OAS2	2.762570290	2.161252380
OAS1	2.807803790	2.457138480
STAT1	3.158411500	2.877180000
IFIH1	3.421704730	2.308702960
ISG15	4.673623260	4.124464650
IFIT3	5.250479150	3.354502550
MX2	5.667869040	4.344987600
IFIT2	5.792369160	3.399964050
IFIT1	6.063912410	3.028201990

Figure S7 (Related to Figure 5). Comparison of the transcriptome and proteome in infected Raji cells. Raji cells were infected with MeV and collected for bulk RNA sequencing (n=3). **(A)** Detection of MeV transcripts in infected cells is shown as normalized counts for each MeV gene compared to uninfected controls. Median and 95% confidence intervals are shown. **(B)** Correlation plot showing the relationship between differentially expressed proteins (y-axis) identified by MS in Figure 5 with differentially expressed transcripts identified by bulk RNA sequencing (x-axis). Log2FC values for transcripts and proteins that were detected in both RNA and protein assays are shown. Simple linear regression was conducted, and the Pearson correlation value was reported on the plot, along with the significance of the correlation. **(C)** Correlation plot demonstrating the relationship between the list of significantly altered proteins (y-axis) and transcripts (x-axis). Hits that were significant in both assays are denoted in the table shown in **(D)**.
