# Supplementary material for: Immunological landscape of human lymphoid explants during measles virus infection
Source: JCI Insight. 2024 Jul 25;9(17):e172261. doi: 10.1172/jci.insight.172261 (PMC11385098; doi:10.1172/jci.insight.172261)

Full unedited gel for Figure 5D

<u>Primary Antibodies</u>

1. Mouse anti-human IFIT3 (1:1000)
2. Rabbit anti-human ISG15 (1:5000)
3. Mouse anti-human b-Actin (1:1000)
4. Mouse anti-GFP (1:1000)

<u>Secondary Antibodies</u>

1. Goat anti-mouse HRP (1:10000)
2. Goat anti-rabbit HRP (1:10000)
3. Goat anti-mouse AlexaFluor 647 (1:2000)

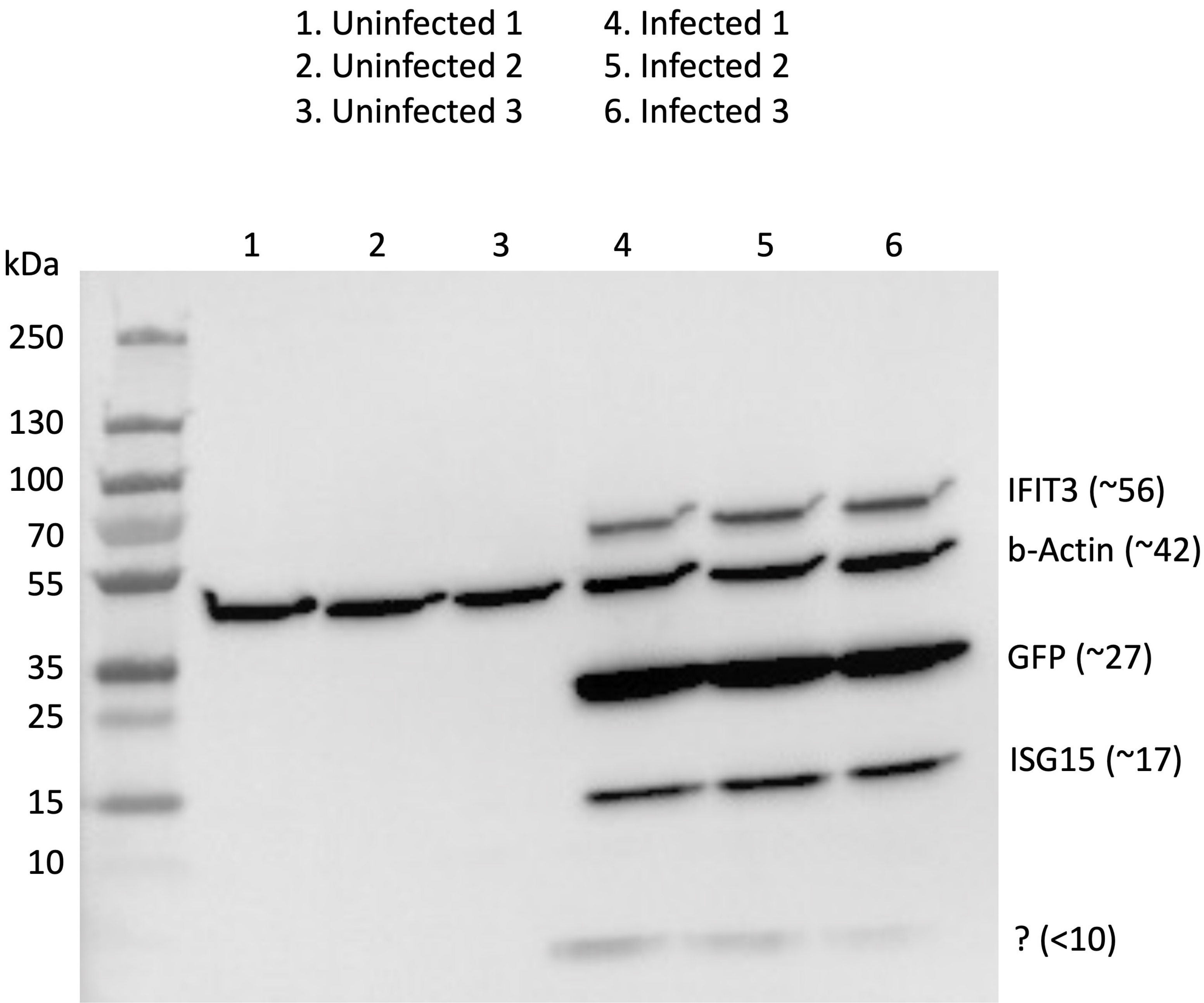

Supplement: Unedited blot and gel images [file jciinsight-9-172261-s120.pdf]
